# RagC senses β-hydroxybutyrate abundancy to suppress mTORC1

**DOI:** 10.1093/procel/pwag017

**Published:** 2026-03-18

**Authors:** Guoyan Wang, Qihang Hou, Yong Zhang, Qiuhui Duan, Jianxuan Gao, Jiangxin Wang, Jinrui Qiao, Yining Zheng, Xinjian Lei, Guowen Liu, Lin Lei, Tong Meng, Xiaojun Yang, Junhu Yao, Xinwei Li, Lu Deng

**Affiliations:** College of Animal Science and Technology, Northwest A&F University, Yangling 712100, China; College of Animal Science and Technology, Northwest A&F University, Yangling 712100, China; Senior Department of General Surgery, The First Medical Center of Chinese PLA General Hospital, Beijing 100853, China; Tongji University Cancer Center, Shanghai Tenth People’s Hospital of Tongji University, School of Life Sciences and Technology, Tongji University, Shanghai 200092, China; Department of Orthopedics, Shanghai General Hospital, School of Medicine, Shanghai Jiaotong University, Shanghai 200092, China; College of Animal Science and Technology, Northwest A&F University, Yangling 712100, China; College of Animal Science and Technology, Northwest A&F University, Yangling 712100, China; College of Animal Science and Technology, Northwest A&F University, Yangling 712100, China; College of Animal Science and Technology, Northwest A&F University, Yangling 712100, China; State Key Laboratory for Diagnosis and Treatment of Severe Zoonotic Infectious Diseases, Key Laboratory for Zoonosis Research of the Ministry of Education, Institute of Zoonosis, and College of Veterinary Medicine, Jilin University, Changchun 130062, China; State Key Laboratory for Diagnosis and Treatment of Severe Zoonotic Infectious Diseases, Key Laboratory for Zoonosis Research of the Ministry of Education, Institute of Zoonosis, and College of Veterinary Medicine, Jilin University, Changchun 130062, China; Department of Orthopedics, Shanghai General Hospital, School of Medicine, Shanghai Jiaotong University, Shanghai 200092, China; College of Animal Science and Technology, Northwest A&F University, Yangling 712100, China; College of Animal Science and Technology, Northwest A&F University, Yangling 712100, China; State Key Laboratory for Diagnosis and Treatment of Severe Zoonotic Infectious Diseases, Key Laboratory for Zoonosis Research of the Ministry of Education, Institute of Zoonosis, and College of Veterinary Medicine, Jilin University, Changchun 130062, China; College of Animal Science and Technology, Northwest A&F University, Yangling 712100, China; Shenzhen Research Institute, Northwest A&F University, Shenzhen 518000, China

**Keywords:** RagC, β-hydroxybutyrylation, mTORC1, tumor growth

## Abstract

The ketogenic diet (KD), an emerging nutritional intervention for cancer, reprograms cellular energy metabolism from glucose to ketone bodies, including acetoacetate (AcAc), acetone (Ac), and β-hydroxybutyrate (BHB). However, the mechanisms connecting ketone body signal sensing to tumor growth suppression remain elusive. Here, we show that RagC, a key component of mTORC1 pathway, senses BHB but not AcAc and Ac, to dictate tumor suppression. KD-derived BHB inhibits mTORC1 activity by promoting β-hydroxybutyrylation (Kbhb) of RagC at lysine 349 (K348 in mice). Mechanistically, RagC-K349bhb is dynamically catalyzed by p300 and erased by SIRT1, disrupting RagC interaction with Raptor/mTOR and blocking mTORC1 recruitment to lysosomes. Clinically, BHB-mediated RagC-K349bhb suppresses colorectal cancer (CRC) growth via mTORC1 inhibition in both RagC-K348R knockin mice and CRC patient-derived samples. Thus, we identify a BHB sensing mechanism by mTORC1 and highlight the potential role of RagC-K349bhb as a therapeutic target for BHB-based CRC treatment.

## Introduction

Sensing the nutrients from the diet is essential for maintaining homeostasis across all organisms (Green et al., 2022). Dysregulation of the sensing machinery underlies metabolic syndromes, neurodegenerative diseases, cancer, et al (Tomita et al., 2020; Goul et al., 2023). The ketogenic diet (KD) is defined as a high-fat, low-carbohydrate, moderate-protein dietary regimen (Corsello et al., 2023). This metabolic intervention promotes hepatic ketogenesis, yielding three primary ketone bodies: acetoacetate (AcAc), acetone (Ac), and β-hydroxybutyrate (BHB), with BHB serving as the predominant circulating energy substrate owing to its stability and abundance (Kang et al., 2015; Newman and Verdin, 2017; Yang et al., 2022). This unique metabolic reprogramming endows KD with potential as an adjuvant strategy for cancer, type 2 diabetes, and polycystic ovary syndrome (Sengupta et al., 2010; Dmitrieva-Posocco et al., 2022; Huang et al., 2024; Madhavan et al., 2025; Moya-Garzon et al., 2025; Roichman et al., 2025). A recent study has indicated that KD-derived BHB, but not AcAc, Ac, is the major metabolite that suppresses the colorectal cancer (CRC) via activation of hydroxy-carboxylic acid receptor 2 (HCAR2) (Dmitrieva-Posocco et al., 2022). However, the precise mechanism by which cellular BHB is sensed remains elusive.

The mechanistic target of rapamycin complex 1 (mTORC1) has been established as one of the major pathways to sense various nutrients, including amino acids, glucose, cholesterol, and fatty acids (Kim and Guan, 2019). Pathological or genetic disruptions in mTORC1 function can lead to aberrant colorectal cell growth, thereby contributing to tumor growth (Brandt et al., 2018; Dai et al., 2022; Solanki et al., 2023). Current understanding highlights that mTORC1 is activated by an array of regulatory molecules in response to fluctuations in amino acid levels and is recruited to the lysosomal surface via the small G protein Rag GTPase (Shen and Sabatini, 2018). Rag GTPase act as a scaffold to interact with regulatory-associated of protein mTOR (Raptor) and facilitate the translocation of mTORC1 to the lysosomal surface in response to amino acid stimulation (Sancak et al., 2008, 2010). Rag GTPase assemble as heterodimeric complexes, wherein the nucleotide-binding state, RagA/B in GTP-bound form and RagC/D in GDP-bound form, enables mTORC1 activation (Sekiguchi et al., 2001). Compelling evidence suggests that the mechanism by which mTORC1 senses nutrients involves the direct binding of nutrients to specific sensors. For instance, amino acids can directly bind specific sensors to regulate mTORC1 (Han et al., 2012; Wang et al., 2015; Chantranupong et al., 2016; Wolfson et al., 2016; Gu et al., 2017; Chen et al., 2021). Recent study also indicates mTORC1 can sense the polyunsaturated fatty acid ω-6 linoleic acid via direct binding to fatty acid binding protein 5 (FABP5) (Koundouros et al., 2025). Beyond direct binding to sensors, emerging evidence has uncovered metabolite-driven post-translational modifications (PTMs) in the mTORC1 pathway as a nutrient/energy-sensing mechanism. For instance, S-Adenosyl methionine methylates NPRL2 (Jiang et al., 2023), acetyl-CoA drives Raptor acetylation (He et al., 2020), and palmitoyl-CoA drives LAMTOR1 palmitoylation (Nada et al., 2009). These findings define metabolite-driven PTMs as a new paradigm that enables mTORC1 to dynamically interpret and respond to nutrient fluctuations.

Sensing of dietary nutrients and energy availability by mTORC1 has been demonstrated to be involved in tumor growth. Although current studies have identified multiple nutrient sensors within the mTORC1 pathway, including sestrin2, leucyl-tRNA synthetase 1 (LRS1), secretion-|associated Ras-related GTPase 1B (SAR1B), CASTOR1/2, and SAMTOR (Han et al., 2012; Chantranupong et al., 2016; Wolfson et al., 2016; Gu et al., 2017; Chen et al., 2021). It is unknown whether Rag GTPases, the core regulators of mTORC1, physically interact with and sense nutrient availability in a manner analogous to established sensors such as sestrin2, LRS1, and SAR1B. Despite evidence that the KD-derived BHB functions as a key regulator of tumor growth (Zheng et al., 2022; Qin et al., 2024), the role of Rag GTPase in BHB sensing and its link to mTORC1 activation and tumor growth remain largely uncharacterized. In this study, we identify a previously unrecognized sensing paradigm where BHB is specifically recognized by RagC through covalent modification, named β-hydroxybutyrylation (Kbhb) (Xie et al., 2016), at lysine 349, thereby inhibiting mTORC1 activity and CRC growth. This discovery of Kbhb-dependent mTORC1 regulatory axis provides unprecedented insights into how dietary metabolites can directly influence tumorigenesis through precise PTM mechanisms.

## Results

### KD suppresses tumor growth via BHB mediated mTORC1 inhibition

**Figure 1.**
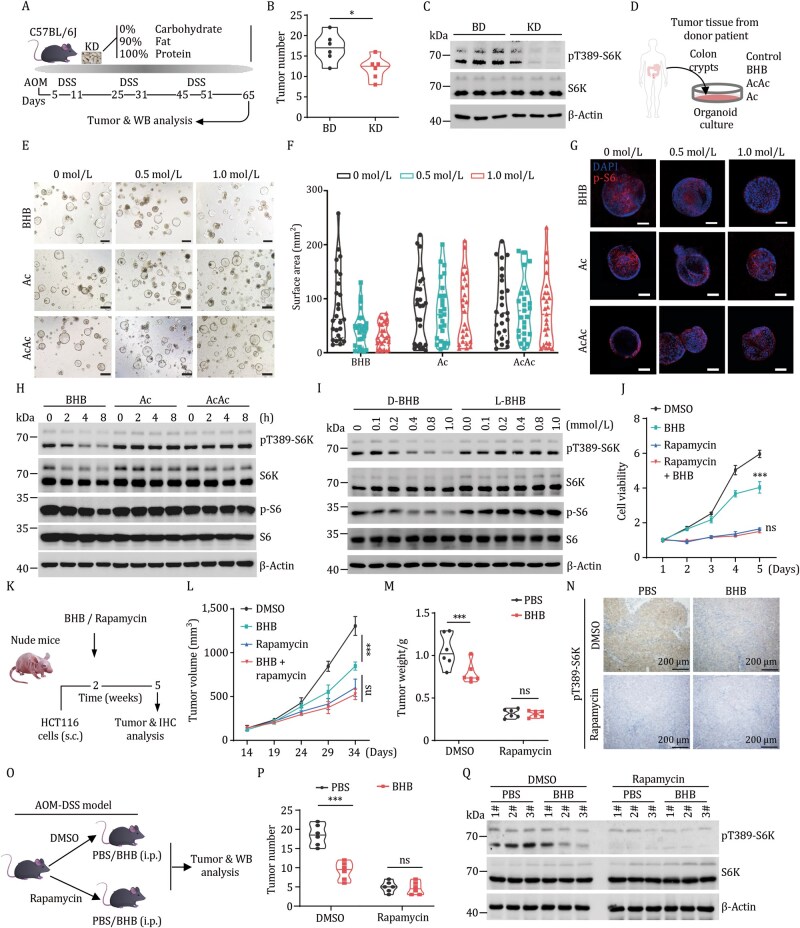
**KD suppresses tumor growth via BHB-mediated mTORC1 inhibition**. (A) Schematic of KD exposure in AOM/DSS-treated mice. (B and C) Tumor number (B) and the levels of pT389-S6K, S6K, Actin in colon tissues (C) of AOM/DSS-treated mice fed KD or baseline diet (BD) were measured. (D) Experimental diagram for analysis of human CRC organoid stimulated with BHB, Ac, and AcAc for 12 h. (E) Representative pictures of human CRC organoid treated with indicated concentrations BHB, Ac, and AcAc for 12 h, scale bar, 500 μm. (F) Graph showing the surface area of organoids in different groups (*n *= 25 per group). (G) Immunofluorescence analysis showed the level of p-S6 in human CRC organoids treated with indicated concentrations of BHB, Ac, and AcAc, scale bar, 100 μm. (H) WB showed the levels of pT389-S6K, S6K, p-S6, S6, and Actin in HCT116 cells treated with 1.0 mmol/L BHB, Ac, and AcAc for different times. (I) WB showed the levels of pT389-S6K, S6K, p-S6, S6, and Actin in HCT116 cells treated with indicated concentrations of D/L-BHB for 12 h. (J) HCT116 cells were treated with 1.0 mmol/L BHB or combined with 100 nmol/L rapamycin and utilized for cell viability assay. (K) Schematic of the CDX experiment subcutaneous injected with HCT116 cells and administered BHB or combined with rapamycin as indicated (*n *= 6 per group). (L–N) The tumor volume (L), tumor weight (M), and IHC of pT389-S6K in tumor tissues (N) of different groups were measured, scale bar, 200 μm. (O) Schematic of BHB and rapamycin exposure in AOM/DSS-treated mice. (P and Q) The number of tumors (P) and levels of pT389-S6K, S6K, and Actin (Q) of different groups were measured. The statistical significance of the differences between groups was determined by (B) unpaired two-tailed Student’s *t* test, (F) one-way ANOVA, or (J, L, M, and P) two-way ANOVA (ns, not significant; *P < 0.05, **P < 0.01, ***P < 0.001). BHB denotes the D-isomer.

The KD has been well-documented to suppress CRC growth (Weber et al., 2020; Dmitrieva-Posocco et al., 2022). KD feeding, consistent with prior findings, elevated plasma BHB levels in an azoxymethane (AOM)/dextran sodium sulfate (DSS) mouse model (Figs. 1A and S1A), and reduced tumor size/burden (Figs. 1B and S1B) relative to control diets. Evidences commonly demonstrate that CRC progression is associated with mTORC1 activation (Wang et al., 2023; Zhao et al., 2024), which encourages us to investigate whether a KD, through its mechanism of inhibiting mTORC1 activity, can suppress CRC growth. Notably, we observed that KD intervention suppressed mTORC1 signaling as indicated by the phosphorylation of p70S6 Kinase 1 (S6K) at Thr389 in colon tumors and normal colon tissue (Figs. 1C, S1C, and S1D), suggesting a potential mechanistic link between KD and mTORC1 inhibition.

The hallmark of KD is the systemic metabolic reprogramming that shifts the primary energy substrate from glucose to ketone bodies (Newman and Verdin, 2017). To further assess the relationship between mTORC1 and ketone bodies, we isolated and cultured mouse colon organoids (Fig. S1E). Among the three ketone bodies—BHB, Ac, and AcAc—only BHB potently suppressed both organoid growth and mTORC1 activity (Fig. S1F–I). Next, we isolated and cultured organoids from human CRC tumor tissues (Fig. 1D). The results showed that only BHB, rather than Ac or AcAc, selectively suppressed the growth as well as the emergence rate of human CRC organoids in a dose-dependent manner (Fig. 1E and 1F). Consistent results were observed in human CRC organoids (Figs. 1G and S1J) and cell models (Figs. 1H and S1K), indicating that only BHB, rather than Ac or AcAc, selectively suppressed mTORC1 activation. Likewise, BHB supplementation in AOM-DSS-derived mouse CRC organoids further validated its inhibitory effects on mTORC1 activity (Fig. S1L and S1M).

BHB exists as D- and L-enantiomers, with endogenous human BHB consisting predominantly (97%–98%) of the D-isomer, while L-BHB has been historically regarded as metabolically inert (Newman and Verdin, 2017). Importantly, our findings revealed that D-BHB (but not L-BHB) inhibited mTORC1 pathway activity in a concentration- and time-dependent manner (Figs. 1I and S2A). Henceforth, all references to BHB in this study denote the D-isomer. In line with human CRC organoids results, BHB inhibited the activity of the mTORC1 pathway in a concentration- and time-dependent manner in multiple CRC cell lines and HEK293T cells (Fig. S2B–I). Furthermore, we found that BHB substantially reduced the viability of CRC cells (Fig. S2J–P). Hence, our findings imply that mTORC1 may sense BHB and be involved in the inhibitory effect of BHB on the growth of colorectal tumor.

To further verify that BHB exerts its tumor suppressor role through the mTORC1 pathway, mTORC1 pathway was stimulated by knocking down TSC2, a negative regulator of mTORC1 (Inoki et al., 2002). Our findings revealed that knockdown of TSC2 significantly abrogated the inhibitory effect of BHB on cell viability (Fig. S3A). In addition, both BHB and rapamycin, an mTORC1 inhibitor, individually reduced cell viability, whereas the inhibitory effect of BHB on cell viability was not aggravated by rapamycin addition (Fig. 1J). Similarly, we found that BHB strongly inhibited the rate of organoid formation and the activity of mTORC1, without showing synergistic effects with rapamycin in a mouse colon organoid model (Fig. S3B–F). These findings indicate that BHB suppresses cell growth by inhibiting the mTORC1 pathway. Moreover, we observed co-treatment with BHB and rapamycin showed no additional benefits over rapamycin alone in terms of tumor volume, tumor weight, or mTORC1 activity in the cell-derived xenograft (CDX) mouse model (Fig. 1K–N). To further validate our observations, we constructed the AOM-DSS mouse model and found both BHB and rapamycin monotherapy reduced tumor number and mTORC1 activity compared to controls, while rapamycin treatment blocked the inhibitory effect of BHB (Fig. 1O–Q). Critically, TSC2 knockdown in nude mice abolished the tumor-suppressive effects of BHB (administered via intraperitoneal injection) and restored mTORC1 activity (Fig. S3G–J). Hence, our findings reveal that BHB, generated by KD, inhibits mTORC1 pathway, thereby suppressing CRC cells’ activity, colon organoid growth, and CRC progression.

### BHB inhibits mTORC1 through RagC β-hydroxybutyrylation (RagC Kbhb)

**Figure 2.**
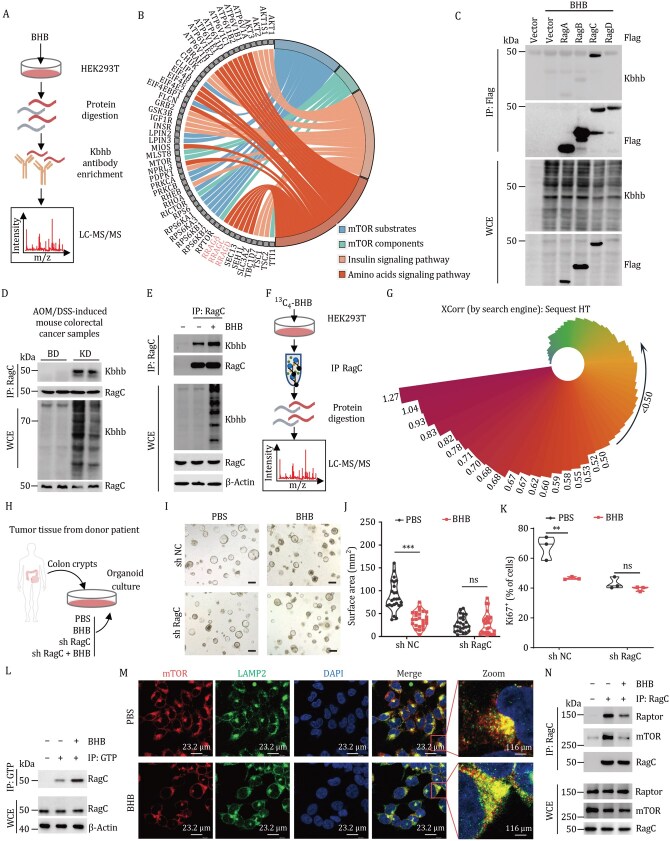
**BHB inhibits mTORC1 through RagC β-hydroxybutyrylation (RagC Kbhb)**. (A) Schematic representation of experimental workflow for the identification of Kbhb-containing protein substrates in HEK293T cells treated with 1.0 mmol/L BHB for 12 h. (B) An enrichment chord plot was performed Kbhb protein involved in the mTOR pathway. (C) Detecting Kbhb of Rags GTPase by WB in HEK293T cells treated with 1.0 mmol/L BHB for 12 h. (D) WB analysis showed the levels of RagC Kbhb in colon tissues of mice fed a KD or BD. (E) Kbhb modification of endogenous RagC in HEK293T cells treated with 1.0 mmol/L BHB for 12 h. (F) Schematic representation of experimental workflow for the identification of RagC Kbhb in HEK293T cells treated with 1.0 mmol/L 13C4-BHB for 12 h. (G) Rose plot was performed on the XCorr: sequest HT of all peptides. (H) Experimental diagram for analysis of human CRC organoid infected with lentivirus that interfered with endogenous RagC expression and stimulated with 1.0 mmol/L BHB. (I) Representative pictures of human CRC organoids in different groups, scale bar, 500 μm. (J) Graph showing the surface area of organoids in different groups (*n *= 25 per group). (K) Flow cytometry analysis of Ki-67^+^ frequency in human CRC organoids (*n *= 3). (L) HCT116 cells were treated with 1.0 mmol/L BHB for 12 h to detect the GTP-bound RagC via GTP beads. (M) HCT116 cells were treated with 1.0 mmol/L BHB for 12 h and then co-immunostained for mTOR and LAMP2 , scale bar, 23.2 μm. (N) IP showing interactions between RagC and Raptor/mTOR treated with 1.0 mmol/L BHB for 12 h in HEK293T cells. The statistical significance of the differences between groups was determined by (J–K) two-way ANOVA (ns, not significant; **P < 0.01, ***P < 0.001). BHB denotes the D-isomer.

We next aimed to investigate the molecular mechanisms by which BHB regulates the mTORC1 pathway. Given that BHB has been found to mediate a PTM known as Kbhb (Qin et al., 2024), we investigated whether BHB regulates mTORC1 through Kbhb modification. Our data showed a significant Kbhb signal induction across proteins of various molecular weights in AOM-DSS mouse model following KD intervention (Fig. S4A). To systematically identify whether proteins of mTORC1 pathway undergo Kbhb modification, we performed liquid chromatography-tandem mass spectrometry (LC-MS/MS) analysis of BHB-treated cells (Fig. 2A). We identified 20,474 Kbhb sites across 5,491 proteins (Fig. S4B–D), with subcellular distribution analysis demonstrating widespread distribution across all major cellular compartments (Fig. S4E). To determine a possible consensus motif for Kbhb, we compared the amino acid sequences surrounding the modification sites by software MoMo based on the motif-x algorithm and we found a notable preference for alanine, lysine, and arginine at most positions, whereas cysteine, proline, and serine were underrepresented (Fig. S4F). To understand the potential regulation by Kbhb, we performed KEGG enrichment analysis on Kbhb proteomic profiling and noticed BHB-induced Kbhb modifications throughout the mTOR signaling pathway (Fig. S4G and S4H). Enrichment analyses further confirmed that modified sites were preferentially clustered on amino acid-sensing hubs critical for mTORC1 activation, especially Rag GTPase (Fig. 2B). Consequently, we observed that overexpression of persistently activated forms of the heterodimers RagA^Q66L^ and RagC^S75N^ significantly abrogated the BHB-induced inhibition of the mTORC1 pathway and decrease of cell viability (Fig. S5A and S5B). Further screening revealed that the observed Kbhb modification was specific to RagC, with no detectable modification on RagA, RagB, and RagD (Figs. 2C and S5C). Additionally, D-BHB, but not its stereoisomer L-BHB, selectively induces Kbhb modification of RagC (Fig. S5D). Thus, BHB may directly target RagC to regulate mTORC1 activation.

Subsequently, we investigated whether RagC underwent Kbhb modification. Our data showed that KD intervention promoted the enrichment of RagC Kbhb in mouse colon tissue (Fig. 2D). Endogenous immunoprecipitation (IP) assays further revealed increased RagC Kbhb in response to BHB stimulation (Fig. 2E). Most conclusively, ^1^³C_4_-BHB metabolic labeling combined with high-resolution mass spectrometry directly mapped ^1^³C_4_-Kbhb modifications to specific lysine residues on RagC (Figs. 2F, 2G and S5E). To establish the functional significance of RagC Kbhb in mTORC1 regulation, we performed RagC knockdown experiment in human CRC organoids. This genetic intervention completely abolished BHB-mediated suppression of organoid growth and mTORC1 signaling (Figs. 2H–K and S5F), establishing RagC as the primary mediator of BHB’s anti-tumor effects.

Previous studies have reported that BHB can be transported into cells via MCT1 (Nguyen et al., 2021). Therefore, we explored the hypothesis that modification of RagC requires the MCT1-mediated BHB transport. This hypothesis was supported by the finding that MCT1 knockdown significantly reduced RagC Kbhb levels (Fig. S5G). RagC, a small G protein, exists in active (GDP-bound) and inactive (GTP-bound) forms (Sekiguchi et al., 2001). Therefore, we next tried to determine whether BHB influence activity of RagC, and the results showed BHB treatment led to a marked accumulation of RagC in its inactive form (Fig. 2L), and MCT1 knockdown significantly restored RagC activity inhibited by BHB (Fig. S5H).

Current models emphasize that mTORC1 activation requires its recruitment to the lysosomal membrane, a process facilitated by interactions between the Raptor and RagC (Sancak et al., 2008, 2010). Notably, we observed that BHB stimulation reduced mTORC1 recruitment to lysosomal membranes (Fig. 2M) and attenuated RagC-Raptor/mTOR binding (Fig. 2N). Consistently, treatment with the MCT1 inhibitor AZD3965 led to the accumulation of mTOR on the lysosomal membrane (Fig. S5I), and MCT1 knockdown significantly enhanced cell viability compared with BHB treatment alone (Fig. S5J). Collectively, our findings demonstrate that BHB promotes RagC Kbhb, which disrupts mTORC1 localization to lysosomes and inhibits its activation, thereby suppressing CRC growth.

### p300 mediates β-hydroxybutyrylation of RagC

**Figure 3.**
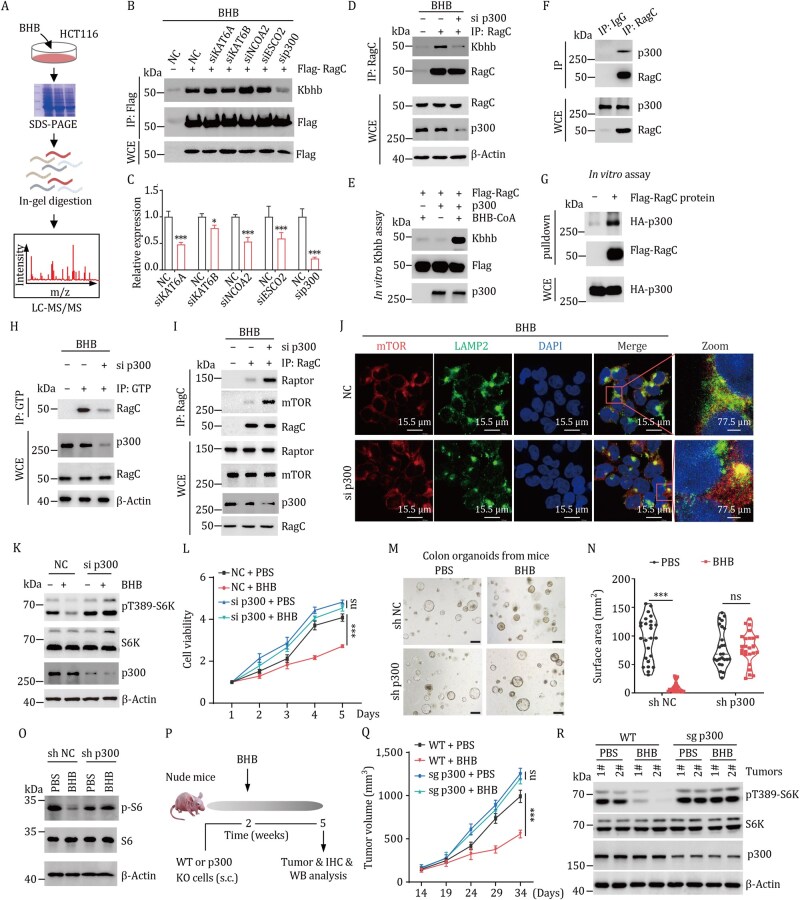
**p300 mediates β-hydroxybutyrylation of RagC**. (A) Schematic of experimental workflow for the identification of proteins that interact with RagC in HCT116 cells treated with 1.0 mmol/L BHB for 12 h using IP-MS. (B) Screening the “reader(s)” of RagC Kbhb by transfecting combined Flag-RagC and indicated acetyltransferases siRNA and treated with 1.0 mmol/L BHB for 12 h. (C) The siRNA knockdown efficiency of the indicated protein was detected by qRT-PCR. (D) Kbhb modification of RagC in HEK293T cells knocked down p300 and treated with 1.0 mmol/L BHB for 12 h. (E) IP RagC proteins were incubated with recombinant p300 protein (100 ng) and β-hydroxybutyryl-CoA (BHB-CoA, 20 μmol/L) for 1 h at 30 °C, pH 8.0. (F) The interaction of endogenous p300 and RagC was analyzed via co-IP assay. (G) *In vitro* pull-down assay indicated that p300 interacts with the Flag-RagC. (H) Detecting the GTP-bound RagC using IP in p300-depletion HEK293T cells treated with 1.0 mmol/L BHB for 12 h. (I) IP showed interaction between RagC and Raptor/mTOR in p300-depleted HEK293T cells treated with 1.0 mmol/L BHB for 12 h. (J) HCT116 cells knocking down p300 were treated with 1.0 mmol/L BHB for 12 h and then co-immunostained for mTOR and LAMP2 , scale bar, 15.5 μm. (K) HCT116 cells knocking down p300 were treated with 1.0 mmol/L BHB for 12 h to detect pT389-S6K, S6K, p300, and Actin levels, NC denotes negative control. (L) HCT116 cells knocking down p300 and treating with 1.0 mmol/L BHB were utilized for cell viability assay. (M) Representative pictures of mouse colon organoids treated with 1.0 mmol/L BHB for 12 h, scale bar, 500 μm. (N) Graph showing the surface area of organoids in different groups (*n *= 25 per group). (O) WB showed the levels of p-S6, S6, and Actin in mouse colon organoids treated with 1.0 mmol/L BHB for 12 h. (P) Schematic of the CDX experiment subcutaneous injected with WT or p300 KO HCT116 cells and administered BHB as indicated (*n *= 6 per group). (Q and R) The tumors volume (Q), and level of pT389-S6K, S6K, and Actin in tumor tissues (R) of different groups were measured. The statistical significance of the differences between groups was determined by (C) unpaired two-tailed Student’s *t* test or (L, N, and Q) two-way ANOVA (ns, not significant; *P < 0.05, ***P < 0.001). BHB denotes the D-isomer.

To identify the enzymes responsible for RagC Kbhb, we performed immunoprecipitation-mass spectrometry (IP-MS) (Fig. 3A), revealing endogenous RagC interactions with 18 acetyltransferases and 14 deacetylases (Fig. S6A). Targeted knockdowns of the top candidate acetyltransferases (lysine acetyltransferase 6A (KAT6A), KAT6B, nuclear receptor coactivator 2 (NCOA2), establishment of sister chromatid cohesion N-acetyltransferase 2 (ESCO2), and p300) revealed that only p300 knockdown or p300 inhibitor treatment significantly reduced RagC Kbhb levels (Figs. 3B–D, S6B, and S6C). *In vitro* assays confirmed recombinant p300 mediates the RagC Kbhb (Fig. 3E). These results implicated p300 as the primary enzyme catalyzing the Kbhb of RagC. Therefore, we next investigated whether a direct interaction exists between RagC and p300. Our IP and pull-down experiments confirmed such a direct interaction (Fig. 3F and 3G). We also assessed p300 activity by monitoring H3K27ac, a recognized marker (Jin et al., 2011). Its levels were unchanged upon BHB treatment, indicating that p300 activity is not affected and ruling out a contribution from this pathway.

We further explored whether p300 affects RagC activity as a RagC-Kbhb catalase. Our results showed that p300 knockdown led to a near disappearance of the GTP-bound form of RagC (Fig. 3H). Then, we examined whether p300 could regulate the binding of RagC to Raptor, thereby influencing mTORC1 activity. Our data show that p300 knockdown significantly enhanced the binding of RagC to Raptor, while p300 overexpression had the opposite effect (Figs. 3I and S6E). In addition, p300 knockdown resulted in increased localization of mTOR on lysosomes (Fig. 3J), indicating that p300 inhibited lysosomal localization of mTOR. Moreover, we found that the decrease in S6K phosphorylation and cell viability induced by BHB can be abrogated by p300 knockdown (Figs. 3K, 3L, and S6F). Similarly, disruption of p300 expression in mouse colon organoids by lentivirus significantly blocked the ability of BHB to inhibit the organoids’ growth (Figs. 3M, 3N, and S6G). This was further corroborated by an increase in S6 phosphorylation (Fig. 3O), indicating enhanced mTORC1 activity upon p300 knockdown. We then constructed p300-knockout cell lines using CRISPR Cas9 and injected them into nude mice subcutaneously (Fig. 3P). We observed that p300 knockout rescues the tumor-suppressive effect of BHB, as measured by tumor volume and weight (Figs. 3Q and S6H). Moreover, we noted that higher mTORC1 activity in the p300-deficient tumors after BHB treatment (Fig. 3R). Collectively, these results suggest that the antitumor activity of BHB is dependent on p300-catalyzed Kbhb of RagC.

### SIRT1 removes β-hydroxybutyrylation of RagC

**Figure 4.**
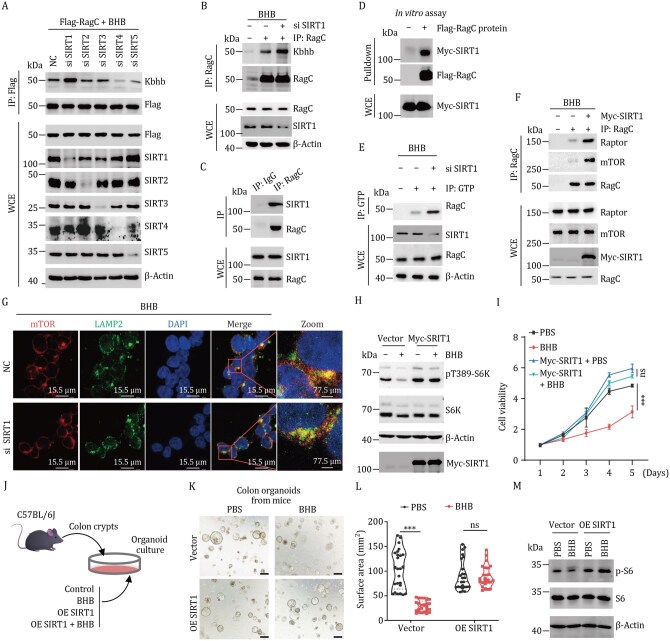
**SIRT1 removes β-hydroxybutyrylation of RagC**. (A) Screening the “eraser(s)” of RagC Kbhb by transfecting combined Flag-RagC and SIRT1-5 siRNA and treating with 1.0 mmol/L BHB for 12 h. (B) Kbhb modification of RagC in SIRT1-knocked down HEK293T cells and treated with 1.0 mmol/L BHB for 12 h. (C) The interaction of endogenous SIRT1 and RagC was analyzed via co-IP assay. (D) *In vitro* pull-down assay indicated that SIRT1 interacts with the Flag-RagC. (E) Detecting the GTP-bound RagC using IP in SIRT1 depletion HEK293T cells treated with 1.0 mmol/L BHB for 12 h. (F) IP showed interaction between RagC and Raptor/mTOR in SIRT1-overexpressed HEK293T cells treated with 1.0 mmol/L BHB for 12 h. (G) HCT116 cells knocking down SIRT1 were treated with 1 mmol/L D-BHB for 12 h and then co-immunostained for mTOR and LAMP2 , scale bar, 15.5 μm. (H) HCT116 cells overexpressing SIRT1 were treated with 1.0 mmol/L BHB for 12 h to detect pT389-S6K, S6K, SIRT1, and Actin levels. (I) HCT116 cells overexpressing Myc-SIRT1 and treating with 1.0 mmol/L BHB were utilized for cell viability assay. (J) Schematic illustration of the experimental diagram for analyzing mice organoids overexpressing SIRT1 and stimulated with 1.0 mmol/L BHB. (K) Representative pictures of mouse colon organoids overexpressed SIRT1 or treated with 1.0 mmol/L BHB, scale bar, 500 μm. (L) Graph showing the surface area of organoids in different groups (*n *= 25 per group). (M) WB showed the levels of p-S6, S6, and Actin in mice colon organoids treated with 1.0 mmol/L BHB for 12 h. The statistical significance of the differences between groups was determined by (I and L) two-way ANOVA (ns, not significant; ***P < 0.001). BHB denotes the D-isomer.

The HDACs and SIRTs family have been implicated in the deacylation of various protein modifications, including Kbhb (Zhang et al., 2019; Huang et al., 2021). Accordingly, we treated cells with TSA (an inhibitor of HDACs) and NAM (an inhibitor of SIRTs) and found that NAM significantly enhanced the level of RagC Kbhb (Fig. S6I), suggesting SIRTs involvement. We then screened the SIRTs that might be involved by specific siRNAs and found that SIRT1 knockdown specifically enhanced the level of RagC-Kbhb, which was confirmed by SIRT1 inhibitor treatment (Figs. 4A, 4B, and S6J). Furthermore, co-IP results revealed a binding between RagC and SIRT1 (Fig. 4C), and pull-down experiments provided further proof of a direct interaction between them (Fig. 4D). These results show that SIRT1 can, indeed, remove the Kbhb modification of RagC. Furthermore, our experimental result confirmed that, similar to p300, SIRT1 activity was likewise unaffected by BHB (Fig. S6K).

To explore the functional consequences of SIRT1-medicated removal of RagC Kbhb, we assessed the activity of RagC. Our result exhibited SIRT1 knockdown resulted in a significant enhancement of the GTP-bound form of RagC (Fig. 4E), suggesting that SIRT1 facilitates RagC activity by removing its Kbhb modification. Notably, we observed SIRT1 knockdown reduced the binding of RagC to Raptor, while overexpression of SIRT1 had the opposite effect (Figs. 4F and S6L). Furthermore, SIRT1 knockdown also resulted in a remarkable decrease in the lysosomal localization of mTOR (Fig. 4G), linking Kbhb to mTORC1 spatial regulation.

We next explored whether SIRT1 affect mTORC1 activity by detecting the phosphorylation of S6K. Our data showed SIRT1 overexpression abrogated the BHB-mediated decreases in S6K phosphorylation and cell viability, while depletion of SIRT1 had the opposite effect (Figs. 4H, 4I, and S6M). Furthermore, these findings were replicated in a mouse colon organoid model that SIRT1 overexpression by lentivirus blocked BHB-induced growth retardation and altered S6 phosphorylation (Figs. 4J–M and S6N). This further verified that SIRT1 opposes the inhibition of mTORC1 activity by BHB. Collectively, these results suggest that the inhibitory effect of BHB on mTORC1 activity is dependent on p300-catalyzed and SIRT1-abolished Kbhb of RagC.

### RagC is β-hydroxybutyrylated at K349

**Figure 5.**
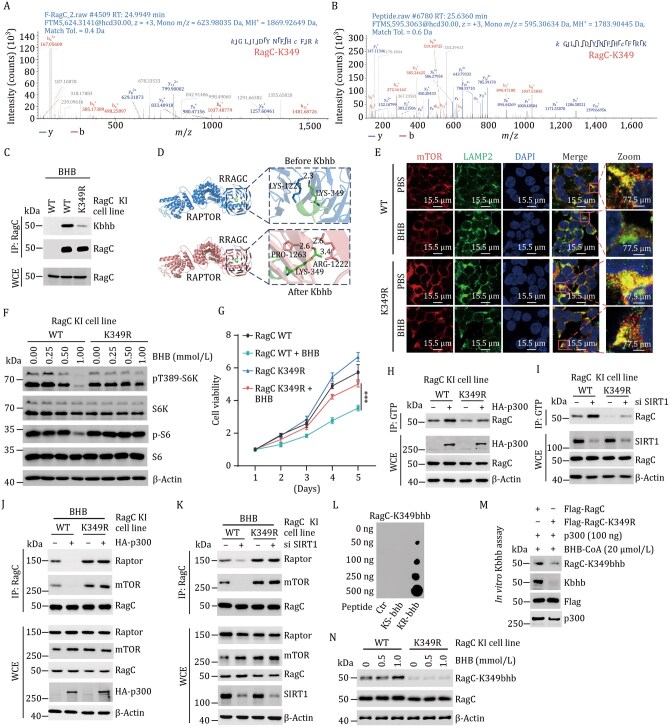
**RagC is β-hydroxybutyrylated at K349**. (A) MS/MS spectra from LC-MS/MS analysis of a β-hydroxybutyrylated peptide (the K349 site of human RagC) derived from HEK293T cells treated by 1.0 mmol/L BHB. (B) MS/MS spectra from HPLC-MS/MS analysis of a β-hydroxybutyrylated peptide (the K349 site of human RagC) derived from *in vitro* generated counterpart (synthetic peptide). (C) Kbhb modification of endogenous RagC in RagC WT or K349R KI HCT116 cells treated with 1.0 mmol/L BHB for 12 h. (D) Structures of wild-type and K349 β-hydroxybutyrylated RagC in the interaction with Raptor from the last snapshot of the MD simulation. (E) RagC WT or K349R KI HCT116 cells were treated with 1.0 mmol/L BHB for 12 h and then co-immunostained for mTOR and LAMP2 , scale bar, 15.5 μm. (F) RagC WT or K349R KI HCT116 cells treated with indicated concentrations of BHB for 12 h were utilized for detecting pT389-S6K, S6K, p-S6, S6, and Actin levels. (G) RagC WT or K349R KI HCT116 cells treated with 1.0 mmol/L BHB were utilized for cell viability assay. (H and I) Detecting the GTP-bound RagC using IP in RagC WT or K349R KI HCT116 cells treated with 1.0 mmol/L BHB for 12 h and combined with HA-p300 overexpression (H) or SIRT1 depletion (I). (J and K) IP showed interaction between RagC and Raptor/mTOR in RagC WT or K349R KI HCT116 cells treated with 1.0 mmol/L BHB for 12 h and combined with HA-p300 overexpression (J) or SIRT1 depletion (K). (L) Dot blot assays defined the specificity of RagC-K349bhb antibody. (M) Detecting RagC-K349bhb using an *in vitro* Kbhb assay as indicated. (N) Detecting RagC-K349bhb in RagC WT or K349R KI HCT116 cells treated with indicated concentrations of BHB for 12 h. The statistical significance of the differences between groups was determined by (G) two-way ANOVA (***P < 0.001). BHB denotes the D-isomer.

To determine the specific Kbhb sites on RagC, we performed high-resolution mass spectrometry analysis and identified six modified residues (K79, K101, K188, K302, K387, and K349) according to peptide confidence and number of secondary mass spectra. Site-directed mutagenesis of these residues to arginine (K→R) revealed that only the K349R substitution abrogated RagC Kbhb, and the K349 site exhibits high conservation among different species (Figs. 5A and S7A–C), implicating this residue as the primary modification site. Metabolic labeling with ^1^³C_4_-BHB and MS definitively mapped the β-hydroxybutyryl group to K349’s ε-amino group, confirming site-specific modification (Fig. S7D). Synthetic peptides encompassing the K349 locus (K_((R)-3-Hydroxybutanoic acid)_ GLIDYNFHCFRK) recapitulated this modification *in vitro* (Fig. 5B), while CRISPR-engineered RagC-K349R knockin (KI) cells exhibited complete loss of detectable Kbhb signals by immunoblot (Fig. 5C).

Subsequent functional studies showed that the GTP-bound form of RagC was reduced in the RagC-K349R KI cells (Fig. S7E). Structural analyses via molecular docking revealed that unmodified RagC-K349 residue forms a stabilizing hydrogen bond (2.3 Å, −0.62 kcal/mol) with Raptor-K1221, whereas K349bhb disrupts this interaction (+0.71 kcal/mol) despite an expanded binding interface (Fig. 5D). Consistent with the structural analysis, RagC-Raptor binding was dramatically strengthened in RagC-K349R cells and became insensitive to BHB stimulation (Fig. S7F). Moreover, BHB also failed to alter mTORC1 lysosomal localization and its activity or cell viability in RagC-K349R cells (Fig. 5E–G), suggesting that K349bhb is the key mediator of BHB-induced mTORC1 suppression. We further validated the role of p300 and SIRT1 and found that neither p300 overexpression nor SIRT1 silencing in RagC-K349R cells altered the effect of BHB on RagC activity and interaction with Raptor (Fig. 5H–K).

To conclusively verify K349’s role in BHB-mediated mTORC1 inhibition, we developed a RagC-K349bhb-specific antibody that recognized wild-type (WT) RagC but not K349R mutant RagC (Fig. S7G), with stereochemical specificity for D-BHB-derived modifications (Fig. 5L). Furthermore, an *in vitro* Kbhb assay directly indicated RagC-K349bhb (Fig. 5M), and dose- and time-dependent BHB treatment increased K349bhb levels in WT but not K349R cells (Figs. 5N and S7H). In summary, our *in vivo* and *in vitro* results conclusively verify that RagC undergoes Kbhb specifically at K349.

### RagC-K349bhb inhibits colorectal tumors in mice

To extend our observations *in vivo*, we produced RagC-K348R (human K349R)-targeted KI mice, and the PCR sequencing results showed that the RagC-K348R KI mouse was constructed successfully (Fig. S8A–C). We found that RagC-K348R KI mice grew faster starting from week 5 and appeared to have longer colons and larger livers (Fig. S8D and S8E). However, histological and hematochemical analysis showed that there is no impact in the liver, kidney function, and BHB levels between WT and RagC-K348R KI mice (Fig. S8F–J).

**Figure 6.**
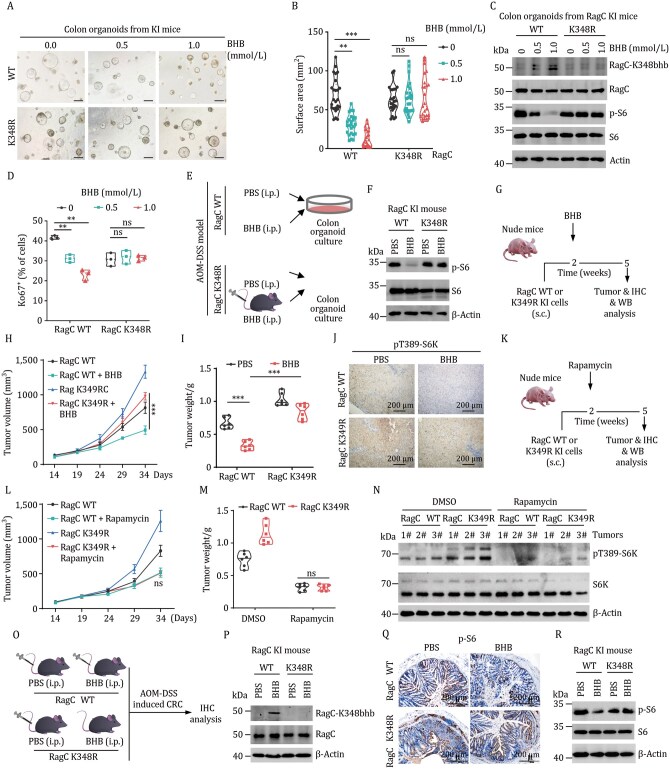
**RagC-K349bhb inhibits colorectal tumors in mice**. (A) Representative picture of mice colon organoids isolated from WT or KI mice treated with 0.0, 0.5, or 1.0 mmol/L BHB for 12 h, scale bar, 500 μm. (B) Graph showing the surface area of organoids in different groups (*n *= 25 per group). (C) WB showed the levels of p-S6, S6, RagC-K349bhb, RagC and Actin in mouse colon organoids treated with 0.0, 0.5, or 1.0 mmol/L BHB for 12 h. (D) Flow cytometry analysis of Ki-67^+^ frequency in mice colon organoids treated with 0.0, 0.5, or 1.0 mmol/L BHB for 12 h (*n *= 3). (E) Schematic of BHB exposure in AOM/DSS-treated RagC WT or KI mice. (F) WB showed the levels of p-S6, S6, RagC-K349bhb, RagC, and Actin in CRC organoids treated with 1.0 mmol/L BHB for 12 h. (G) Schematic of the CDX experiment subcutaneously injected with RagC WT or K349R KI HCT116 cells and administered BHB as indicated (*n *= 6 per group). (H–J) The tumor volume (H), tumor weight (I) and level of pT389-S6K in tumor tissues (J) in different groups were measured, scale bar, 200 μm. (K) Schematic of the CDX experiment subcutaneous injected with RagC WT or K349R KI HCT116 cells and administered rapamycin as indicated (*n *= 6 per group). (L–N) The tumors volume (L), tumors weight (M) and level of pT389-S6K, S6K, and Actin in tumor tissues (N) of different groups was measured. (O) Experimental diagram for AOM/DSS-treated RagC WT or K348R KI mice with intraperitoneal injection BHB. (P–R) The level of RagC-K349bhb (P) and p-S6 (Q and R) in tumor tissues of different groups was measured. The statistical significance of the differences between groups was determined by (B, D, H, I, L, and M) two-way ANOVA (ns, not significant; **P < 0.01, ***P < 0.001). BHB denotes the D-isomer.

Next, we isolated and cultured organoids from RagC-WT and RagC-K348R KI mice. In RagC-WT organoids, we observed that the increasing BHB concentrations led to a gradual increase in RagC-K348bhb levels and a marked decrease in mTORC1 activity, whereas no such changes were detected in RagC-K348R KI organoids (Fig. 6A–D). To clarify the regulatory role of RagC-K349bhb in mTORC1 activation within CRC, we found that BHB injection suppressed S6 phosphorylation in WT-derived CRC organoids, whereas p-S6 levels showed no response to BHB injection in K348R KI-derived CRC organoids (Figs. 6E, 6F, and S8K). In addition, data from CDX experiments confirmed that BHB effectively inhibited tumor growth and mTORC1 activity, yet this suppressive effect was markedly abrogated by the RagC-K349R mutation (Fig. 6G–J). We investigated the role of mTORC1 in rapamycin-treated nude mice (Fig. 6K). The results showed that rapamycin consistently inhibited tumor formation in both RagC-WT and K349R cell lines, accompanied by a suppression of mTORC1 activity (Fig. 6L–N).

We further validated the role of RagC-K349bhb in the KD-mediated anti-tumor effect and found that, compared with the control diet, the KD diet reduced tumor number and mTORC1 activity in the AOM/DSS-induced WT mice model (Fig. S8L–N), and increased RagC-K348bhb levels (Fig. S8O). However, this effect of KD was completely abolished in RagC K348R KI mice (Fig. S8M–O), indicating that BHB-mediated RagC-K348bhb modification is essential for KD-induced mTORC1 inhibition and subsequent tumor growth suppression. To further establish that BHB suppresses mTORC1 activity through RagC-K349bhb in colorectal cancer, we employed an AOM-DSS-induced colorectal cancer model comparing WT and K348R KI mice (Fig. 6O). Our findings demonstrated that BHB markedly increased the level of RagC-K348bhb in mice colorectal tumors, whereas RagC-K348bhb was not detectable in RagC K348R KI mice (Figs. 6P and S8P). Additionally, BHB inhibited mTORC1 activity, as measured by the p-S6, this inhibitory effect, however, was abrogated in RagC K348R KI mice (Fig. 6Q and 6R). Collectively, these findings demonstrate that the BHB-induced inhibition of mTORC1 activity is contingent upon the RagC Kbhb modification specifically at the K349 site.

### Inhibition of human CRC requires RagC-K349bhb

**Figure 7.**
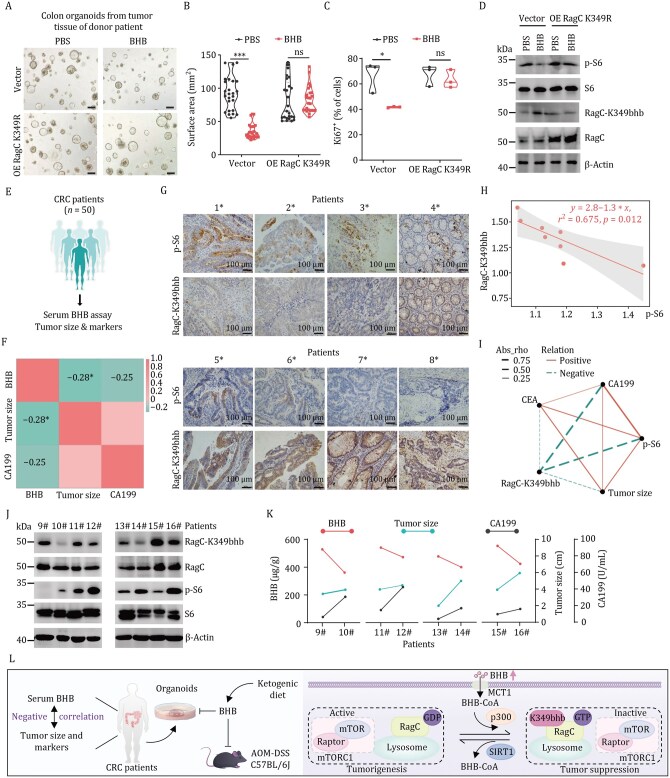
**Inhibition of human CRC requires RagC-K349bhb**. (A) Representative picture of human CRC organoids treated with 1.0 mmol/L BHB for 12 h, scale bar, 500 μm. (B) Graph showing the surface area of human CRC organoids treated with 1.0 mmol/L BHB for 12 h (*n *= 25 per group). (C) Flow cytometry analysis of Ki-67^+^ frequency in human CRC organoids treated with 1.0 mmol/L BHB for 12 h (*n *= 3). (D) WB showed the levels of p-S6, S6, RagC-K349bhb, RagC, and Actin in human CRC organoids treated with 1.0 mmol/L BHB for 12 h. (E) Schematics overview of the correlation analysis performed on samples from CRC patients. (F) Heatmap displaying the correlations between plasma BHB levels and tumor size or CA199 in CRC patients. (G) Representative IHC pictures of p-S6 and RagC-K349bhb in CRC patients, scale bar, 100 μm. (H) Linear regression model between p-S6 and RagC-K349bhb. (I) Correlation network displaying p-S6, RagC-K349bhb, tumor size, CEA, and CA199 in CRC patients. (J) WB analysis showed the levels of RagC-K349bhb, RagC, p-S6, S6, and Actin in CRC patients. (K) A line graph showing the D-BHB level in tumor tissues, tumor size, and CA199 of CRC patients. (L) Mode of inhibition of colorectal cancer by K349bhb of RagC. The statistical significance of the differences between groups was determined by (B and C) two-way ANOVA (ns, not significant; *P < 0.05, ***P < 0.001). BHB denotes the D-isomer.

To validate whether the above observations can be replicated in human cases, we isolated and cultured organoids from human CRC tumor tissues, and the results showed that ectopic expression of RagC-K349R abrogated BHB-mediated suppression of organoid proliferation and restored mTORC1 activity, as evidenced by sustained Ki67 and p-S6 (Fig. 7A–D). Next, we initially investigated the plasma BHB levels in CRC patients and delved into their interrelation (Fig. 7E). Plasma BHB levels were negatively correlated with tumor size and markers (Fig. 7F), implying BHB does exert an inhibitory effect on CRC. Next, we analyzed the relationship between mTORC1 activity and RagC-K349bhb in CRC tumor tissues from patients. The immunohistochemistry (IHC) results unveiled a significant inverse correlation between RagC-K349bhb levels and mTORC1 activity (Fig. 7G and 7I). Of note, the level of p-S6 positively correlated with tumor size and tumor markers (CA199, CEA), while RagC-K349bhb showed the opposite trend (Fig. 7I). In addition, western blot confirmed this antagonistic pattern (Fig. 7J), and lower tissue BHB levels were associated with larger tumors and elevated CA199 in the patients with CRC (Fig. 7K). These findings establish BHB as a potent inhibitor of CRC progression via RagC-K349bhb-dependent mTORC1 suppression and highlight its therapeutic potential (Fig. 7L).

## Discussion

The KD induces metabolic reprogramming by redirecting energy metabolism from glucose to the ketone body BHB. This metabolic reconfiguration renders metabolic disorders—including cancers—more susceptible to standard therapies, thereby highlighting its potential as a promising adjuvant for metabolic disease management (Nencioni et al., 2018; Caffa et al., 2020). Consistent with prior evidence, we demonstrated that KD-derived BHB potently suppresses CRC growth. Importantly, we unveiled an unanticipated regulatory paradigm: BHB, a distinctive energy substrate in ketogenic metabolism, is sensed by RagC via promoting the Kbhb modification, defining its role as an atypical metabolic signaling metabolite. This discovery uncovers a fundamental duality in nutrient sensing wherein ketone metabolites function as critical negative regulators of anabolic pathways.

In this study, we identified a previously unrecognized physiological function of RagC, a core component of the mTORC1 signaling axis, as a sensor of ketone body metabolite BHB derived from KD diets. Notably, BHB induces p300-catalyzed (and SIRT1-reversible) K349bhb of RagC, which disrupted RagC-Raptor/mTOR binding and mTORC1 lysosomal localization, ultimately inhibiting mTORC1 activity. This mechanism differs fundamentally from conventional mTORC1 nutrient sensing, which typically involves non-covalent interactions with adaptor proteins such as sestrin2, SAR1B, CASTOR1/2, Bcl-2-associated athanogene 2, and Adenosine 5′-monophosphate (AMP)-activated protein kinase (Inoki et al., 2003; Chantranupong et al., 2014, 2016; Gu et al., 2017; Chen et al., 2021, 2025). Although we found that BHB affected RagC activity (GTP-bound state), the underlying mechanism remains unclear. It is classically understood that FLCN complex, comprising folliculin (FLCN) and FLCN-interacting proteins 1 and 2 (FNIP1/2), functions as a GAP for RagC (Tsun et al., 2013; Lawrence et al., 2019). Therefore, further investigation will be important to determine how the K349bhb modification influences FLCN complex association or induces structural rearrangements within RagC’s nucleotide-binding domains. Overall, our study highlights a nutrient-sensing inhibitory mechanism where BHB suppresses mTORC1 via RagC Kbhb modification, positioning RagC as a central BHB sensor that integrates metabolic cues into cellular signaling networks. While our data establish RagC-K349bhb as a key regulatory mechanism, we cannot exclude potential roles for other Rag GTPases, as RagA/D were also detected by MS, and weak WB signals were observed for RagA/B/D (Fig. 2B and 2C). Future work to precisely map modification sites on RagA/B/D will be valuable. Interestingly, a recent study showed that KD or BHB supplementation modified the Kbhb of aldolase B, a non-component protein of the mTORC1, which indirectly inhibits mTORC1 (Qin et al., 2024). Together, these findings indicate BHB may regulate mTORC1 via multiple substrates Kbhb modification.

Precedent studies of BHB-mediated protein modifications used D/L-BHB mixture (Xie et al., 2016; Huang et al., 2021; Dmitrieva-Posocco et al., 2022; Qin et al., 2024). Importantly, our study demonstrated that D-BHB, but not L-BHB, mediates RagC covalent modification, enabling mTORC1 pathway recognition. Previous investigations of BHB function have predominantly employed supraphysiological concentrations (5–50 mmol/L) (Liu et al., 2019; Huang et al., 2021; Koronowski et al., 2021; Dmitrieva-Posocco et al., 2022; Li et al., 2022; Zheng et al., 2022; Han et al., 2025), whereas physiological levels range from 100 to 250 μmol/L under normal conditions and reach around 1 mmol/L after 24-h fasting (Koronowski et al., 2021). Notably, KD intervention elevates circulating BHB to only 2–3 mmol/L (Qin et al., 2024), which is lower than BHB concentrations used in previous literatures. To better mimic physiological relevance, we restricted BHB to less than 1 mmol/L in all experimental systems. Strikingly, our results show that even at a physiological concentration of 1 mmol/L BHB induced by a ketogenic diet, rather than at supraphysiological levels (5–50 mmol/L), exerts significant inhibitory effects on mTORC1 activity and tumor growth, with this process being dependent on RagC-K349bhb. These results reveal the critical role of KD-mediated RagC-K349bhb in cancer therapy.

Consistent with preclinical evidence that KD exerts anticancer effects via unique metabolic rewiring (Salvadori et al., 2021; Dmitrieva-Posocco et al., 2022), our study shows that BHB suppresses mTORC1 activation and CRC growth by RagC K349bhb. Importantly, the RagC-K349/348R KI abrogates this inhibitory effect during CRC growth, highlighting the critical role of RagC Kbhb in mediating BHB’s antitumor activity. Furthermore, we observed an inverse correlation between RagC K349bhb levels and CRC progression in patients. These findings indicate that RagC K349bhb demonstrates therapeutic potential in CRC models. Additionally, KD has potential therapeutic effects for type 2 diabetes, aging, and neurodegenerative disorders (Sengupta et al., 2010; Madhavan et al., 2025; Moya-Garzon et al., 2025), a comprehensive understanding of the role of RagC K349bhb in other KD-associated pathological contexts will likely provide mechanistic insights into multiple human diseases.

Overall, our findings establish RagC as a BHB sensor that undergoes Kbhb at lysine 349, a modification catalyzed by p300 and reversed by SIRT1. Significantly, we detected an inverse correlation between RagC K349bhb levels and CRC progression in patient samples, indicating that RagC K349bhb harbors therapeutic potential in CRC models. Our study illuminates a distinctive regulatory framework for cellular adaptation to ketone metabolism, underscoring RagC’s unique role as a nutrient (BHB, an alternative energy substrate) sensor with profound physiological and pathological implications.

## Supplementary Material

pwag017_Supplementary_Data

## Data Availability

The datasets generated during and/or analyzed during the current study are available from the corresponding author on reasonable request.

## Abbreviations

Ac: acetone
AcAc: acetoacetate
AOM: azoxymethane
BHB: β-hydroxybutyrate
CDX: cell-derived xenograft
CRC: colorectal cancer
DSS: dextran sodium sulfate
ESCO2: establishment of sister chromatid cohesion N-acetyltransferase 2
FABP5: fatty acid binding protein 5
FLCN: folliculin
FNIP1/2: FLCN-interacting proteins 1 and 2
HCAR2: hydroxy-carboxylic acid receptor 2
IF: immunofluorescence
IHC: immunohistochemistry
IP: immunoprecipitation
IP-MS: immunoprecipitation-mass spectrometry
KAT6A: lysine acetyltransferase 6A
Kbhb: β-hydroxybutyrylation
KD: ketogenic diet
KI: knockin
KO: knockout
LC-MS/MS: liquid chromatography-tandem mass spectrometry
LRS1: leucyl-tRNA synthetase 1
mTORC1: mechanistic target of rapamycin complex 1
NC: nitrocellulose
NCOA2: nuclear receptor coactivator 2
PTMs: post-translational modifications
qRT-PCR: RNA extraction and quantitative reverse transcription polymerase chain reaction
Raptor: regulatory-associated of protein mTOR
RIPA: radio immunoprecipitation assay
S6K: p70S6 Kinase 1
SAR1B: secretion-associated Ras-related GTPase 1B
SDS-PAGE: sodium dodecyl sulfate-polyacrylamide gel electrophoresis
siRNA: small interfering RNA
WT: wild-type.
