# Supplementary material for: RagC senses β-hydroxybutyrate abundancy to suppress mTORC1
Source: Protein Cell. 2026 Mar 18;17(8):759–74. doi: 10.1093/procel/pwag017 (PMC13429462; doi:10.1093/procel/pwag017)
Supplement: pwag017_Supplementary_Data [file pwag017_supplementary_data.pdf]

MATERIALS AND METHODS

Cell culture and treatment

HEK293T, the colon cancer cell lines HCT116, HT-29, SW480, DLD-1, Caco-2, LoVo cells were cultured in DMEM supplemented with 10% FBS at 37°C in 5% (v/v) CO2. For western blot assays, cells were usually treated with AcAc (HY112540B, MedChemExpress), acetone (Ac), D-BHB (298360, Sigma-Aldrich), and L-BHB (H3145, Sigma-Aldrich) at concentrations of 0.0, 0.5 and 1.0 mM, NAM (S1899, Selleckchem) at a concentration of 10 mM, TSA (HY-15144, MedChemExpress) at a concentration of 50 nM for 12 h. All BHB utilized thereafter denotes the D-isomer. For immunofluorescence (IF) assays, cells were usually treated with BHB at concentrations of 1.0 mM. For CCK-8 assays, cells were usually treated with targeted with BHB at concentrations of 0.5 or 1.0 mM, rapamycin at a concentration of 100 nM.

Generation of RagC K348R knockin (KI) mice model

RagC K348R KI mice were generated based on the C57BL/6J strain using CRISPR/Cas9 by Cyagen Biosciences in Jiangsu.

Azoxymethane (AOM)/Dextran Sodium Sulfate (DSS) mice model

To induce colitis-associated colon cancer, mice were intraperitoneally injected once with 10 mg/kg body weight of the mutagen AOM (A5486, Sigma-Aldrich), followed by three cycles of 2.0% DSS (160110, MP Biomedicals) in the drinking water for 6 d. Each DSS cycle was followed by regular drinking water for 14 d. Mice were monitored for weight loss daily to assess disease progression. Meanwhile, mice were fed a ketogenic diet (KD), intraperitoneally injection with BHB (15 mg per mouse, once a day) alone, or combined with rapamycin (8 mg/kg, once a day).

Cell-derived xenograft (CDX) mouse model

To test the effect of BHB, approximately 5×10^6 WT or TSC2 depletion, WT or p300 knockout (KO), RagC WT or K349R KI HCT116 cells in 100 μL were subcutaneously transplanted into the flank of 6 to 8-week-old male athymic nude mice. After 14 days, mice with tumors of 40-60 mm^3 were randomly divided into two groups. One group (n = 6) was treated with freshly prepared BHB (15 mg per mouse, once a day) or rapamycin (8 mg/kg, once a day) by intraperitoneal injection. Control group mice (n = 6) were treated with PBS with the same dosing schedule. Tumors were obtained on the 35th day after injection. Tumor sizes were measured with electronic calipers, and volumes calculated using the formula in an unblinded manner: $(L \times W^2) \times 0.5$, where L is length and W is width. All the animal-based experiments were approved by the Institution Animal Care and Use Committee of the Northwest A&F University (NWAFU-2020-1131).

BHB treatment *in vivo* and measurement

Mice were administered with BHB, 200 μL by intraperitoneal injection daily, 15 mg per mouse. BHB was measured in mouse serum. Blood was obtained by orbital vein bleed and was collected into a heparin-coated tube. Serum was separated by centrifugation at 1,000 g for 10 min at 4 °C. Serum samples were frozen at -80 °C until thawed for BHB measurements using a colorimetric assay.

Mouse colon organoid isolation and culture

Mouse colon organoid isolation and culture was largely performed according to our previous study (Hou et al., 2020). Briefly, the normal colon or CRC tissues were collected, opened longitudinally and cut into small pieces. Crypts were isolated following incubating in colon crypt isolation buffer prepared according to the protocols (Ordóñez-Morán, 2020) with 0.5 mM DTT (3483-12-3, Sigma-Aldrich) and 5 mM EDTA (E1170-100, Solarbio). Isolated crypts were suspended in Matrigel (356255, Corning) and then added to the wells of a 24-well plate. IntestiCult™ Organoid Growth Medium (K2204, bioGenous) supplemented with 1% penicillin and streptomycin (SV30010, Hyclone) was added to culture organoid and replaced every 3-4 d.

Human CRC organoid isolation and culture

All adult human serum sample and tumor tissues were obtained from the Chinese PLA General Hospital. All experiments were reviewed and approved by the review board of the Ethics Committee of the Chinese PLA General Hospital (S2022-148-01). All patients undergoing resection of a CRC tumor at the Chinese PLA General Hospital were approached and asked if they would be willing to participate in a CRC biobanking study in which a blood sample and extra tumor tissue would be collected. Human CRC organoids were isolated from fresh human CRC tissues. Human CRC organoids were cultured in 50 μL Matrigel drops in human colorectal cancer organoid medium.

Organoid treatment

Intestinal organoids from mice or humans were treated with BHB, Ac or AcAc at concentrations of 0, 0.5 and 1.0 mM for 12 h. The organoid size was quantified using ImageJ. Rapamycin was added to organoid cultures at a concentration of 100 nM.

Flow cytometric assessment of organoid proliferation

For Ki-67 staining, organoids were dissociated into single cells using trypsin. Then cells were fixed, permeabilized, stained with Ki-67 antibody and subsequently stained with DyLight 488 (ab150080, Abcam)-conjugated secondary antibodies. Samples were detected with a BD FACSCanto™ II (BD) and analyzed with FlowJo software.

Lentivirus construction and organoids infection

For generation of lentivirus, HEK293T cells were plated on 6-well plates in high-glucose DMEM with addition of 3-4 μg plasmid DNA (cloned guides), with 2 μg psPAX2 and 1 μg pMD2.G plasmids mixed into 400 μL PBS to generate lentiviral particles. The packaging vectors were co-transfected using 8 μL PEI (23966, Polysciences) per well. After 5 h, the culture medium was replaced with DMEM. After 24 h and 48 h, the viral supernatants were collected. Organoids transfection with lentivirus was performed as previously reported (Maru et al., 2016). Briefly, colon organoids were dissociated into single cells using TrypLE (12605028, Thermo Fisher Scientific). The organoid cell suspensions were resuspended with transfection mix (300 μL complete DMEM F12 + 8 μg/mL polybrene + 50 μL viral supernatants) and added to 24-well plates pretreated with Matrigel. After 8 h of infection, the mix medium was discarded, followed by the addition of Matrigel and covered with 500 μL of new medium. Organoid growth was analyzed over the next 3–5 days, and the

efficiency of p300 and RagC knockdown, SIRT1 and RagC-K349R overexpression was measured by western blot.

Generation of RagC K349R KI and p300 KO cell lines

RagC K349R KI and p300 KO cells were generated using CRISPR/Cas9 and specific sgRNA oligos were designed by the CRIPSR website (http://crispr.mit.edu/). The targeting sequence at K349 was 5'-GAAGAAAGCTTTGAAAGAAA-3' and the oligo donor sequences was K349R: 5'-GTCTGCATTCTAAGGGAAGAAAGCTTTGAAAGAAGAGGTAATAGCATTTTAAAAAGCTGTTTTTATGTTAATTTCT-3'. For p300, the sgRNA sequence was TCAACCTGGAGCTCTCAACC. Then, 2 μg sgRNA and 2 μg donor DNA were transfected into HCT116 cells. 24 h after transfection, positive cells were screened with puromycin (2 μg/mL). Finally, PCR was performed (F': 5'-TAGGTTAAAGGAAGATGG-3'; R': 5'-GACAAAAGCAGTTTGAGC-3') and the products were sequencing to identify RagC K349R. While, p300 knockout was detected using western blot.

Cell viability

Cells were seeded in 96-well plates at a concentration of 5×10^3 cells per well with or without BHB treatment at the indicated concentrations for the indicated time. Cell viability was measured using CCK-8 kit (C6005L, UElandy) according to the manufacturer's instructions. Briefly, 10 μL reagent was added to each well and incubated at 37°C for 2-3 h. Then the plates were scanned with a plate reader at 450 nm (TECAN, Spark™, Switzerland). For some experiments, rapamycin was added in addition to BHB.

RNA extraction and quantitative reverse transcription polymerase chain reaction (qRT-PCR) analysis

Total RNA was extracted using TRIzol and reverse transcribed using the HiScript II 1st Strand cDNA Synthesis Kit (R212-02, Vazyme), and qRT-PCR was performed with the ChamQ Universal SYBR qPCR Master Mix (Q711-02, Vazyme) on an Applied Biosystems CFX96 machine. The $2^{-\Delta\Delta CT}$ method was used for relative quantification of gene expression after normalization to β-actin expression. Primer sequences are shown in Table S1.

Small interfering RNA (siRNA) knockdown

Non-specific control siRNA and siRNAs for p300, sirtuin (SIRT)1-5, establishment of sister chromatid cohesion N-acetyltransferase 2 (ESCO2), nuclear receptor coactivator 2 (NCOA2), lysine acetyltransferase 6A (KAT6A) and KAT6B were purchased from GenePharma (Shanghai, China) (Table S2). Cells were transfected with siRNA oligonucleotides using Lipofectamine 2000 (11668027, Thermo Fisher Scientific). siRNA transfection of cells was performed according to the manufacturer's instructions.

Co-immunoprecipitation (Co-IP) and western blot

Co-IP and western blot were performed as previously described (Deng et al., 2019). Transfected HEK293T cells were lysed with lysis buffer (containing NP-40) at 4°C for 30 min, then centrifuged in a microcentrifuge at 12,000 rpm for 15 min to remove cellular debris, and incubated with anti-Flag-agarose beads (A2220, Sigma-

Aldrich) for 2-3 h. Endogenous RagC was immunoprecipitated using anti-RagC polyclonal antibody (H00064121-A01, Novus Biologicals). The beads were washed extensively and boiled for immunoblotting detection. For binding of RagC to GTP-agarose beads (G9768, Sigma-Aldrich), cells were harvested on approximately 90% confluency, suspended in binding buffer (0.1% TritonX-100 and a cocktail of proteinase inhibitors) and lysed using three freeze-thaw cycles, then centrifuged at 14,000 g. The supernatants were incubated with 50 μL of GTP-agarose suspension for 2-3 h with rotation at 4°C. The beads were pelleted by centrifugation, washed three times in binding buffer and suspended in 40 μL sample buffer for immunoblotting. For tissue samples, they were suspended in Radio Immunoprecipitation Assay (RIPA) buffer for grinding and holding on ice for 30 min. Proteins were separated by sodium dodecyl sulfate-polyacrylamide gel electrophoresis (SDS-PAGE) and transferred to nitrocellulose (NC) membranes (0.45 μm, GE). Then the membrane was then probed with primary and secondary antibodies. Western blot analysis was performed using commercial antibodies at a 1:1,000 ratio for GAPDH (db106, Diagbio Biosciences), β-actin (66009-1-Ig, Proteintech), RagC (26989-1-AP, Proteintech), Raptor (20984-1-AP, Proteintech), RagA (4357, Cell Signaling), pT389-S6K (9234, Cell Signaling), S6K (9202, Cell Signaling), mTOR (2972, Cell Signaling), p-S6 (4858, Cell Signaling), S6 (2217, Cell Signaling), TSC2 (4308, Cell Signaling), p300 (86377, Cell Signaling), SIRT1 (9475, Cell Signaling), SIRT2 (A3967, ABclonal), SIRT3 (A20805, ABclonal), SIRT4 (A7585, ABclonal), SIRT5 (A23083, ABclonal), Acetyl-Histone H3 (Lys27) (8173, Cell Signaling), MCT1 (HA722556, HuaBio), Flag tag (db7002, Diagbio Biosciences), HA tag (db2603, Diagbio Biosciences), Myc tag (16286-1-AP, Proteintech), Pan-Kbhb (PTM-1201RM, PTM Biolab). After being incubated with primary antibody overnight, the membrane was incubated with horseradish peroxidase (HRP)-conjugated secondary antibody for 1 h at room temperature, and finally the proteins were detected using imaging system (Bio-Rad, Hercules, CA, USA).

Liquid Chromatography-Mass Spectrometry (LC-MS/MS) analysis of β-hydroxybutyrylation (Kbhb) proteomics and RagC Kbhb

For proteomics analysis, HEK293T cells were treated with 1 mM BHB for 12 h. Then, the proteins extracted from HEK293T cells were subjected to in-solution tryptic digestion. Enrichment of Kbhb peptides was carried out by IP with pan anti-Kbhb antibody, which were analyzed by LC-MS/MS. To identify RagC Kbhb sites, HEK293T cells were treated with 1 mM BHB or $^{13}C_4$-BHB (HY-W015851S2, MedChemExpress) for 12 h before harvest and then lysed. The lysates were purified using anti-RagC. The pellet was then resolved on SDS–PAGE and stained with Coomassie blue staining. The band corresponding to RagC was excised and sent for mass spectrometry analysis (BD FACSAria™ III).

RagC Kbhb *in vitro*

RagC proteins was enriched by anti-Flag-agarose beads, and then p300 proteins (81093, Active motif) or BHB-CoA (H0261, Sigma-Aldrich) were added in reaction buffer (50 mM tris-HCl [pH 8.0], 10% glycerol, 100 mM EDTA, 1 mM DTT, and 1 mM phenylmethylsulfonyl fluoride [PMSF]). The mixtures were incubated at 30°C

for 1 h, followed with detection of the levels of Kbhb and K349bhb (PTM Biolab) by western blot.

Immunohistochemistry (IHC)

The tissue specimens were fixed overnight in 4% paraformaldehyde and then were dehydrated in increasing concentrations of isopropyl alcohol, followed by clearing of alcohol by xylene. The specimens were subsequently embedded in paraffin wax in cassettes for facilitation of tissue sectioning. For immunohistochemistry, tissue sections were deparaffinized and incubated in citrate buffer at 95°C for 40 min for antigen retrieval and then incubated overnight at 4°C with the primary antibodies including anti-pT389-S6K (1:50 dilution), and anti-p-S6 (1:50 dilution), anti-RagC-K349bhb (1:50 dilution), after which streptavidin–horseradish peroxidase conjugates were added and the slides incubated for 45 min. After three washes with PBS, DAB solution was added and the slides were counterstained with hematoxylin.

IF

The cells and intestinal organoids were washed three times with PBS and fixed with 4% paraformaldehyde at room temperature for 15 min and permeabilized with 0.2% Triton X-100 for 20 min on ice. Following permeabilization, cells and intestinal organoids were blocked with 1% BSA for 30 min at room temperature and then incubated with specific primary antibodies overnight at 4°C. After washing three times with PBS, cells were incubated with secondary antibodies (Alexa Fluor 488–conjugated anti–mouse IgG (A21202), Alexa Fluor 488–conjugated anti–rabbit IgG (A21206) or Alexa Fluor 555–conjugated anti–mouse IgG (A31570) for 1 h. Nuclei were counterstained with DAPI. All images were collected with a confocal microscope.

***In vitro* pull-down assay**

The Flag-RagC was transfected into HEK293T cells and immunoprecipitated using anti-Flag-agarose beads. The HA-p300 or Myc-SIRT1 protein was incubated with Flag-RagC in CHAPS lysis buffer (40 mM HEPES, pH 7.4, 120 mM NaCl, 1 mM EDTA, 10 mM β-glycerophosphate, 0.3% CHAPS, and a cocktail of proteinase inhibitors) for 2 h, after extensive washing, the beads were boiled, and the bound HA-p300 or Myc-SIRT1 was detected via western blot.

SIRT1 activity measurement

HEK293T cells were treated with 0.5 or 1.0 mM BHB for 12 h and SIRT1 activity was examined using a SIRT1 Activity Assay Kit (Abcam, ab156065) according to the manufacturer's instruction. Data were obtained as relative fluorescence and expressed as fold change relative to the control condition.

Statistical analysis

For cell assays, data are represented as the mean ± S.D of three independent experiments. For the animal study, data are represented as the mean ± S.D of 6 mice. Statistical analyses were performed in GraphPad Prism with the student's t-test, one-way and two-way ANOVA.

Table S1 Primer sequences for qRT-PCR

Gene	Forward primer sequence (5'→3')	Reverse primer sequence (5'→3')

p300	TGCCAAACCAGATGATGCCT	ATAGCCCATAGGCGGGTTGA
KAT6A	AGATTGGCCCACAGACAATCA	GACTTGTGGATCAGGGGGAC
KAT6B	CAACAACAGGGGGACACAAAA	TTAACTGGGAAATCAAGACCAGA
NCOA2	TCACCTCTAGGTCACCCTCC	CACATATCAGCAACTGTGCCT
ESCO2	GACCAGCTCATCATCGACGC	TGTGGCAGAACCAACACGAT
β-Actin	AAGGACCTCTACGCCAACACG	TTTGCGGTGGACGATGGAG

Table S2 The target sequences of si/shRNA for knockdown

Gene	5'→3'
si MCT1	GTATAGTCATGATTGTTGGTGG
si SIRT1	GAAGTTGACCTCCTCATTG
si SIRT2	GCCATCTTTGAGATCAGCTAT
si SIRT3	TTGCTGCATGTGGTTGATTTC
si SIRT4	GAACCCTGACAAGGTTGATTT
si SIRT5	GAGATCCATGGTAGCTTATTT
si p300	CUAGAGACACCUUGUAGUATT
si KAT6A	ATGCCTACTCCCGCCTATAAT
si KAT6B	CCCAAACGTATGCGTCGTAAA
si NCOA2	ATCCGTTCTCAGACTACTAAT
si ESCO2	CAGAAGCAAACAGGCAAATTT
sh TSC2	CACTGGCCTTGGACGGTATTG
sh RagC	CCGGCCTGTGGATATGCAATCTTATCTCGAGATAAGATTG CATATCCACAGGTTTTTG

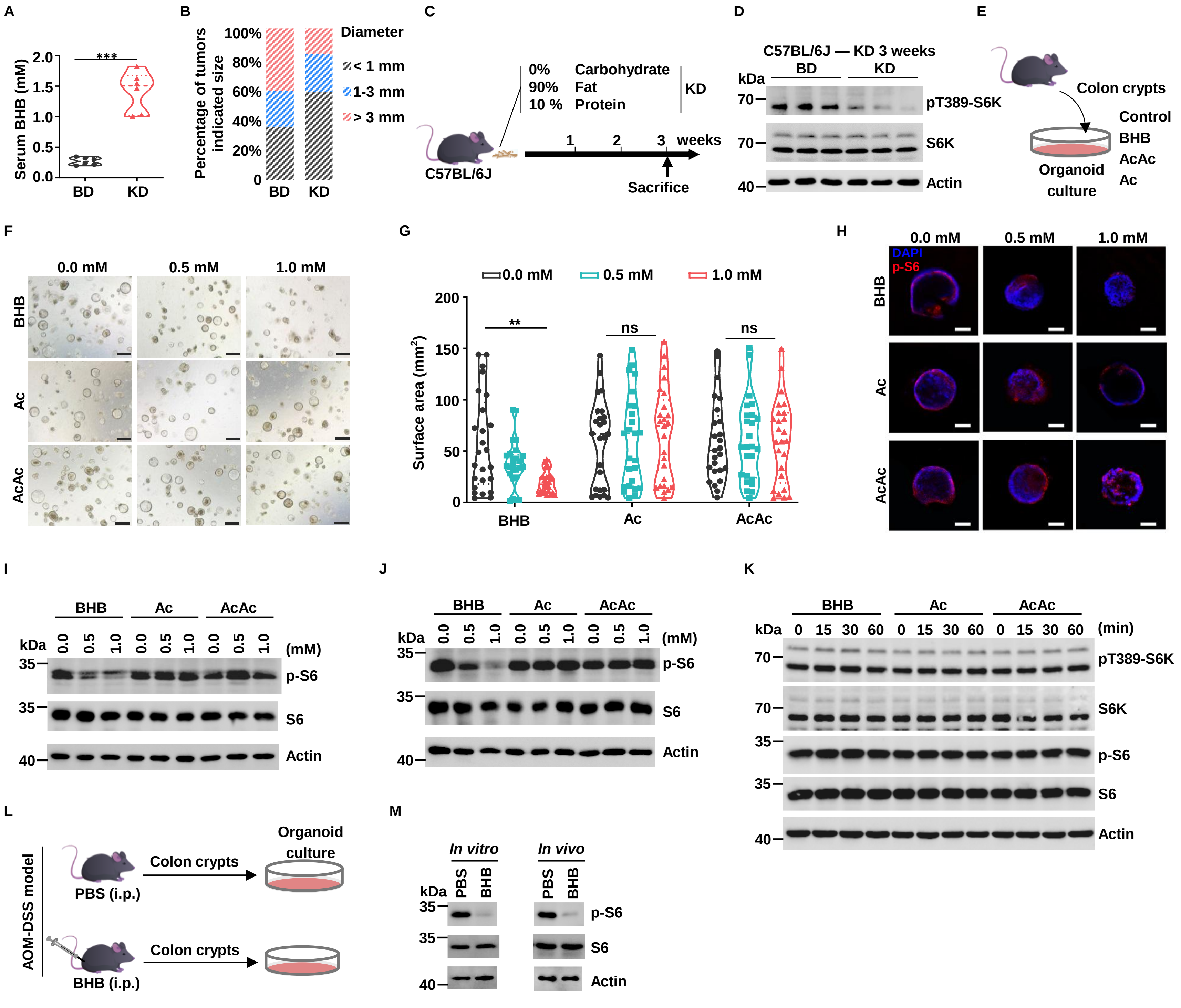

Figure S1. KD-derived BHB inhibits mTORC1 activity.

(**A**) Serum concentrations of BHB in AOM/DSS-treated mice fed KD (n = 6 per group). (**B**) The tumors number of AOM/DSS-treated mice fed KD or control diet were measured. (**C**) Schematic of KD exposure in C57BL/6J mice. (**D**) WB analysis showed the levels of pT389-S6K, S6K, and Actin in colon tissues of mice fed a KD or control diet (BD). (**E**) Experimental diagram for analysis of mice colon organoid stimulated with indicated concentrations of BHB, Ac and AcAc for 12 h. (**F**) Representative pictures of mice colon organoid in different groups, scale bar, 500 μm. (**G**) Graph showing the surface area of organoids in different groups (n = 25 per group. (**H** and **I**) IF (H) and WB (I) analysis showed the level of p-S6 in mice colon organoids in different groups, scale bar, 100 μm. (**J**) WB analysis showed the levels of p-S6, S6, and Actin in human CRC organoid treated with indicated concentrations BHB, Ac and AcAc. (**K**) WB showed the levels of pT389-S6K, S6K, p-S6, S6, and Actin in HCT116 cells treated with 1.0 mM BHB, Ac and AcAc for different time. (**L**) Experimental diagram for CRC organoids from AOM-DSS-treated-mice stimulated with/without BHB *in vitro*, and colon organoids from normal mice with intraperitoneal injection of BHB or PBS *in vivo*. (**M**) WB analysis showed the levels of p-S6, S6, and Actin in different groups.

The statistical significance of the differences between groups was determined by (A) unpaired two-tailed Student's t test or (G) one-way ANOVA (ns, not significant; ***p < 0.001). BHB denotes the D-isomer.

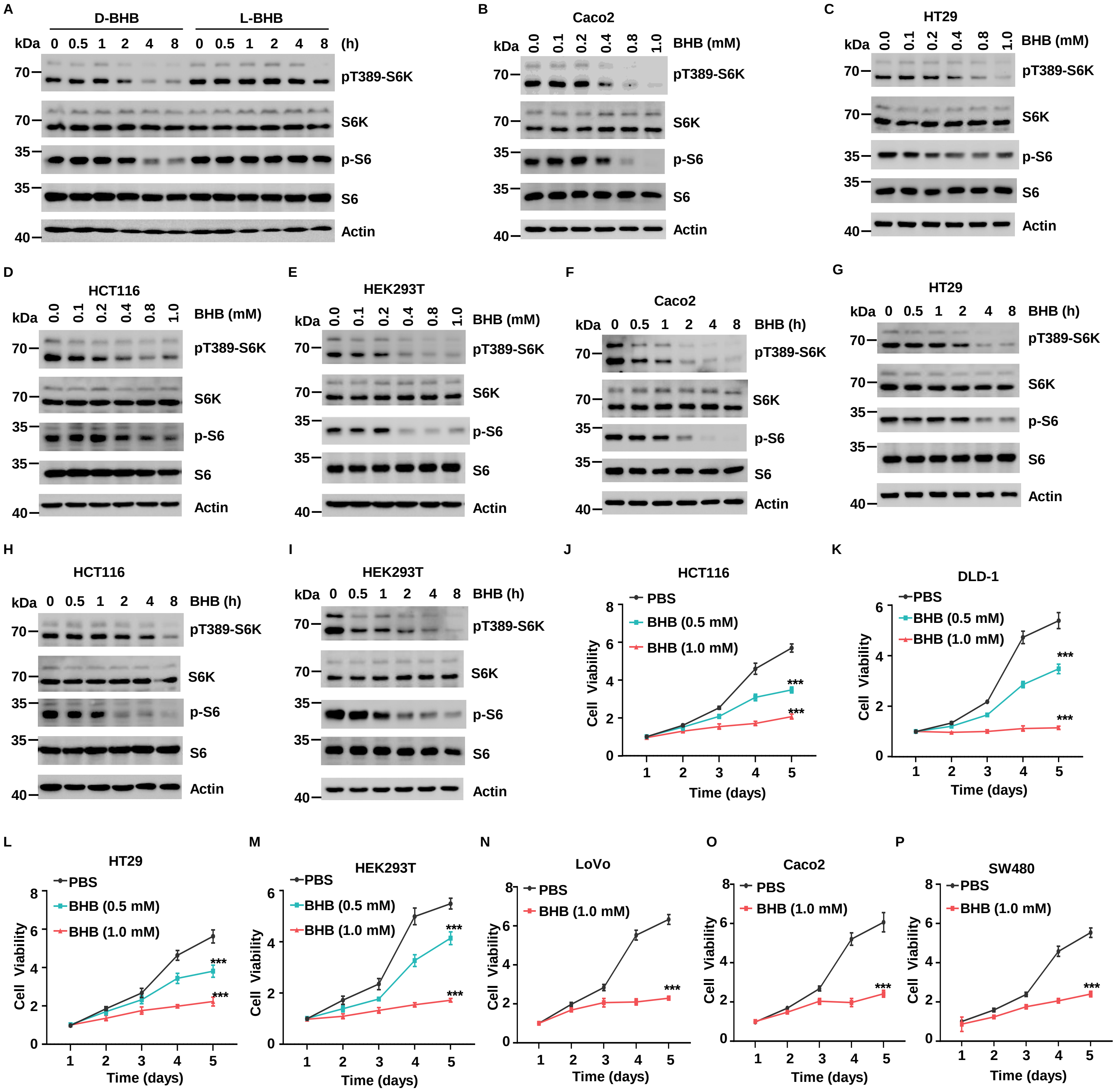

Figure S2. BHB inhibits mTORC1 activity in multiple cell lines.

(**A**) WB showed the levels of pT389-S6K, p-S6 and indicated proteins in HCT116 cells treated with 1.0 mM D/L-BHB for indicated time. (**B** to **E**) WB showed the levels of pT389-S6K, S6K, p-S6, S6, and Actin in Caco2 (B), HT29 (C), HCT116 (D), and HEK293T (E) cells treated with indicated concentrations of BHB for 12 h. (**F** to **I**) WB showed the levels of pT389-S6K, S6K, p-S6, S6, and Actin in Caco2 (F), HT29 (G), HCT116 (H), and HEK293T (I) cells treated with 1.0 mM BHB for indicated time. (**J** to **P**) HCT116 (J), DLD-1 (K), HT29 (L), HEK293T (M), LoVo (N), Caco2 (O), and SW480 (P) cells were treated with indicated concentrations of BHB and utilized for cell viability assay.

The statistical significance of the differences between groups was determined by (J to P) two-way ANOVA (***p < 0.001). BHB denotes the D-isomer.

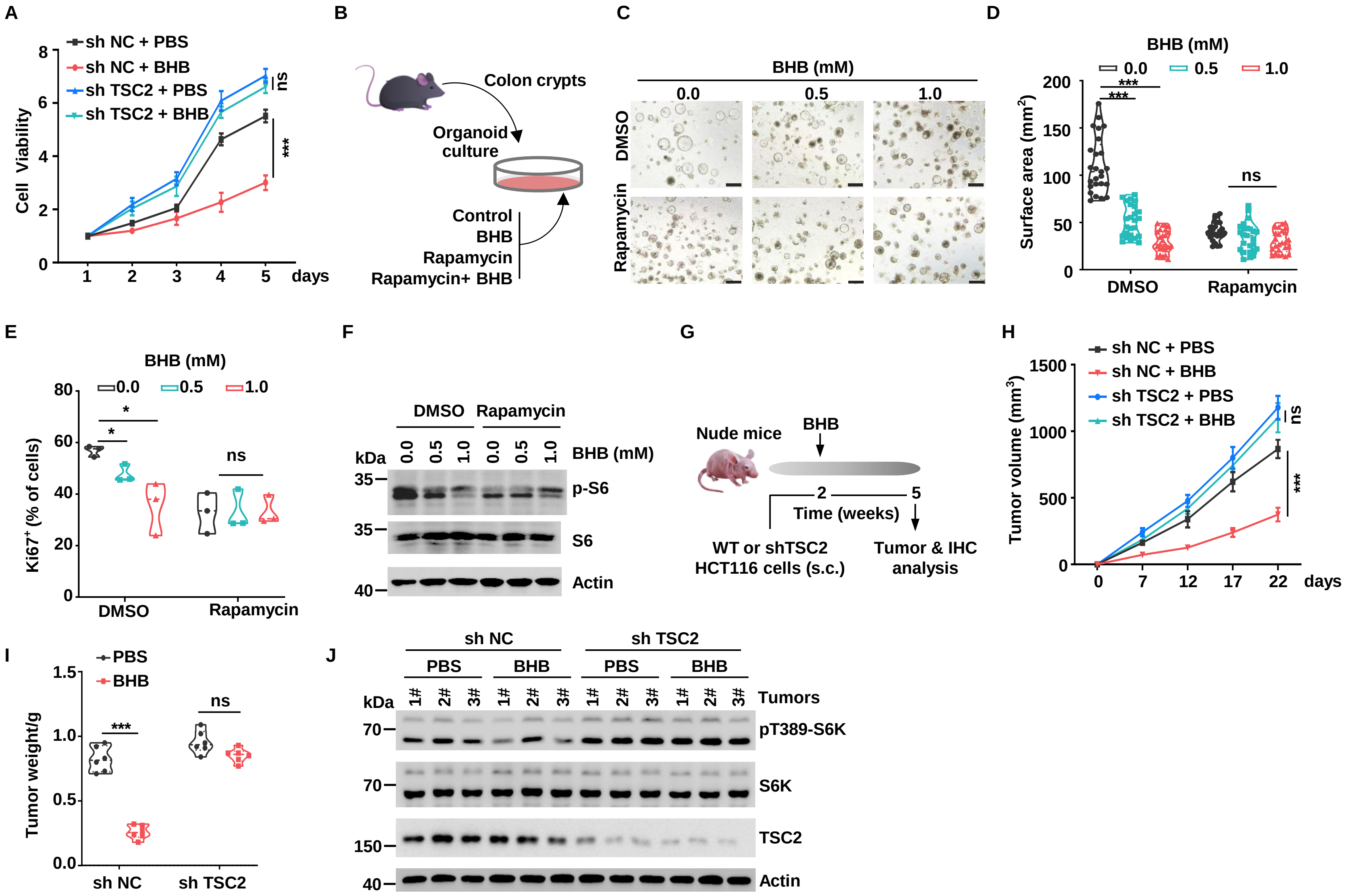

Figure S3. BHB suppresses tumor growth via mTORC1 inhibition.

(**A**) HCT116 cells were treated with 1.0 mM BHB or combined with TSC2 depletion and utilized for cell viability assay. (**B**) Experimental diagram for analysis of mice colon organoid stimulated with BHB or combined with 100 nM rapamycin. (**C**) Representative pictures of mice colon organoids treated with 0.0, 0.5, or 1.0 mM BHB for 12 h, scale bar, 500 μm. (**D**) Graph showing the surface area of organoids in different groups (n = 25 per group). (**E**) Flow cytometry analysis of Ki-67$^+$ frequency in mouse colon organoids treated with 0.0, 0.5, or 1.0 mM BHB for 12 h (n = 3). (**F**) WB showed the levels of p-S6, S6, and Actin in mice colon organoid treated with 1.0 mM BHB or combined with 100 nM rapamycin for 12 h. (**G**) Schematic of the CDX experiment subcutaneous injected with HCT116 cells and administered BHB as indicated (n = 6 per group). (**H** to **J**) The tumors volume (H), tumors weight (I) and levels of pT389-S6K, S6K, TSC2, and Actin in tumor tissues (J) of different groups were measured.

The statistical significance of the differences between groups was determined by (A, D to E, and H to I) two-way ANOVA (ns, not significant; *p < 0.05, ***p < 0.001). BHB denotes the D-isomer.

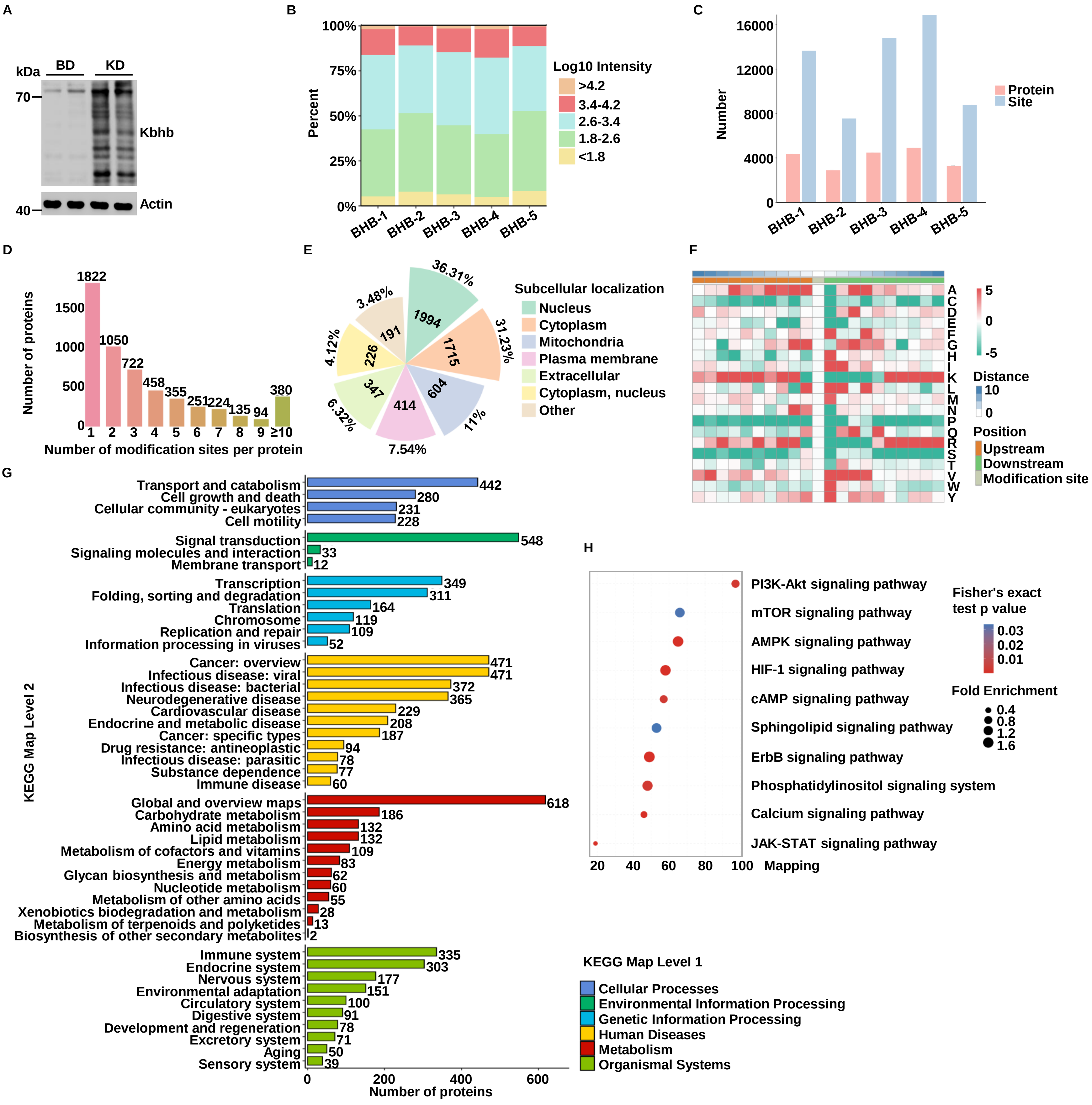

Figure S4. Kbhb exists in mTOR signaling pathway.

(**A**) WB analysis showed the levels of Kbhb in colon tissues of mice fed a KD or control diet. (**B**) Bar chart was performed the intensity distribution of each sample. (**C**) Bar chart was performed the number of modified proteins and sites identified in each sample. (**D**) Distribution of the Kbhb sites based on the site number per protein. (**E**) The number of Kbhb proteins and their subcellular distribution. (**F**) Motif analysis by software MoMo based on motif-x algorithm. (**G**) Results from KEGG map classify analysis of identified proteins. (**H**) Bubble plot was performed the signaling pathway from signal transduction.

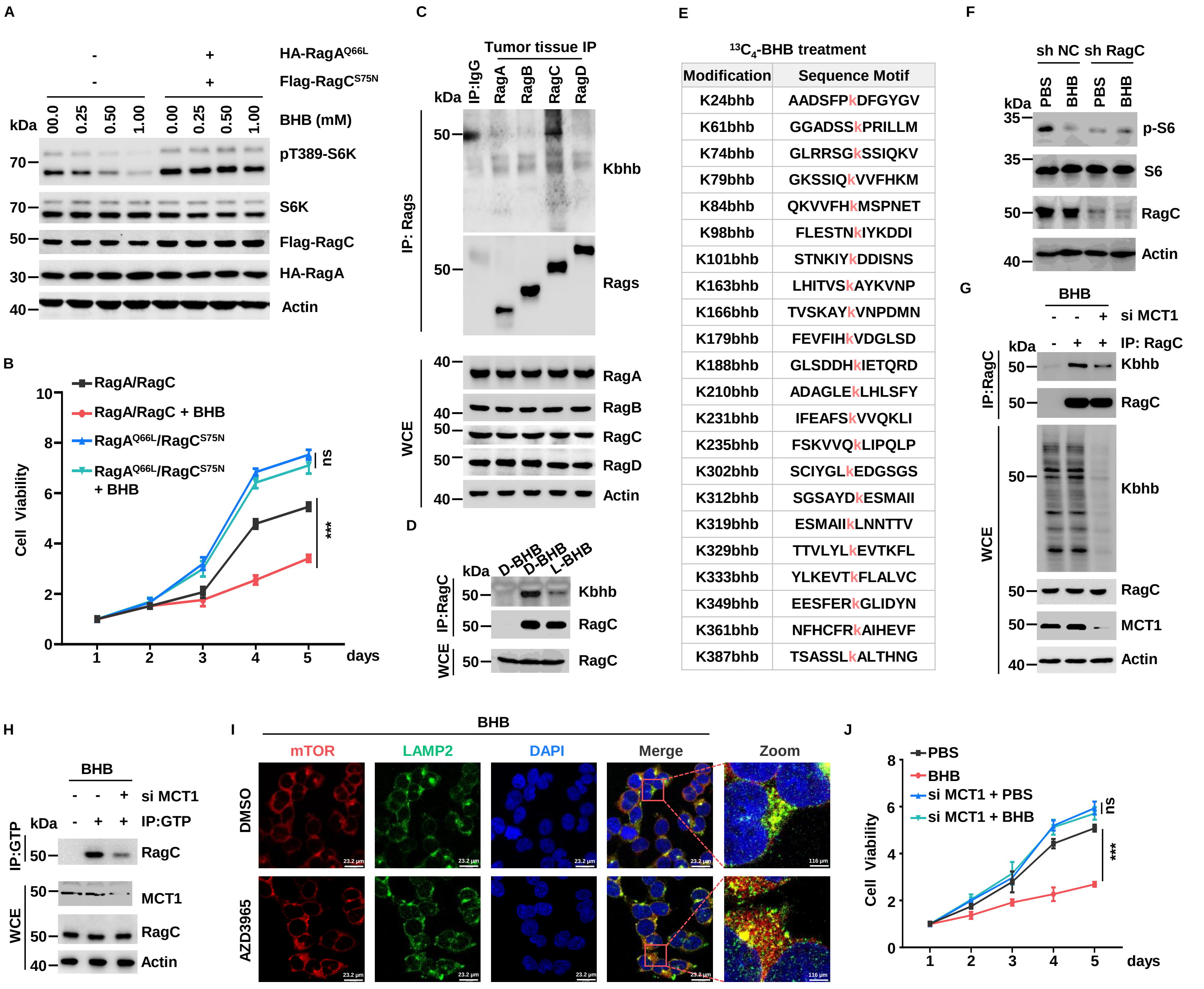

Figure S5. BHB induces RagC β-hydroxybutyrylation (RagC Kbhb).

(**A** and **B**) HCT116 cells transfecting with WT or persistently activated forms of the heterodimers $RagA^{Q66L}$ and $RagC^{S75N}$ and treating with indicated concentrations of BHB were utilized for WB (A) and cell viability assay (B). (**C**) Detecting Kbhb of Rags GTPase by WB using IP tumor tissue sample as indicated. (**D**) Kbhb modification of endogenous RagC in HEK293T cells treated with 1.0 mM D/L-BHB for 12 h. (**E**) The peptides of RagC Kbhb identified by $^{13}C_4$ D-BHB, red, Kbhb site. (**F**) Human CRC organoid infected with lentivirus that interfered with endogenous RagC expression and stimulated with 1.0 mM BHB, WB detected the levels of p-S6, S6, and Actin in different groups. (**G**) Kbhb modification of endogenous RagC in HEK293T cells treated with BHB for 12 h or combination with MCT1 siRNA. (**H**) Detecting the GTP-bound RagC using IP in HEK293T cells treated with 1.0 mM BHB for 12 h alone or combination with MCT1 siRNA. (**I**) HCT116 cells treating with MCT1 inhibitor AZD3965 and then co-immunostained for mTOR (red) and LAMP2 (green), scale bar, 15.5 μm. (**J**) HCT116 cells depleting MCT1 and treating with indicated concentrations of BHB were utilized for cell viability assay.

The statistical significance of the differences between groups was determined by (B and J) two-way ANOVA (ns, not significant; ***$p < 0.001$). BHB denotes the D-isomer.

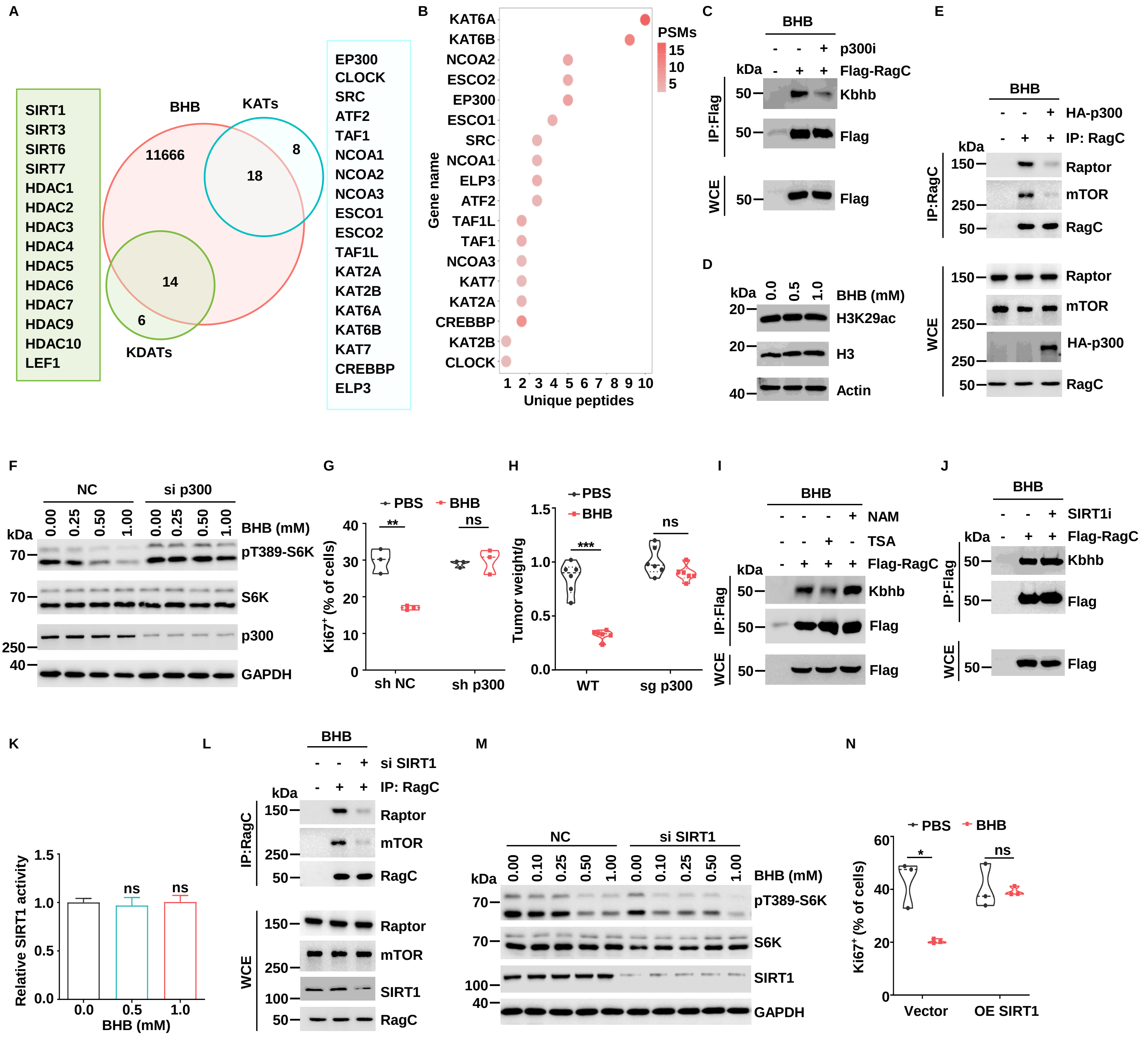

Figure S6. p300 and SIRT1 regulates β-hydroxybutyrylation of RagC.

(**A**) The overlap between IP-MS data from RagC and acetyltransferases/deacetylases. (**B**) Bubble diagram was performed on acetyltransferases. (**C**) Kbhb modification of RagC in HEK293T cells treated with p300 inhibitor (p300i) and 1.0 mM BHB for 12 h. (**D**) H3K29ac levels in HCT116 cells treated with indicated concentrations of BHB for 12 h. (**E**) IP showed interaction between RagC and Raptor/mTOR in HA-p300-overexpressed HEK293T cells. (**F**) WB showed the levels of pT389-S6K, p-S6 and indicated proteins in NC or p300 depleted HCT116 cells treated with indicated concentrations of BHB for 12 h. (**G**) Knocking down p300 in mouse colon organoids, and flow cytometry analysis of Ki-67^{+} frequency in mouse colon organoids treated with 1.0 mM BHB for 12 h (n = 3). (**H**) CDX experiment subcutaneous injected with WT or p300 KO HCT116 cells and administered BHB as indicated (n = 6 per group), the tumors weight were measured. (**I**) Kbhb modification of Flag-RagC in HEK293T cells treated with 1.0 mM BHB and combination with 10 μM NAM or 50 nM TSA for 12 h. (**J**) Kbhb modification of RagC in HEK293T cells treated with SIRT1 inhibitor (SIRTi) and 1.0 mM BHB for 12 h. (**K**) SIRT1 activity in HCT116 cells treated with indicated concentrations of BHB for 12 h. (**L**) IP showed interaction between RagC and Raptor/mTOR in SIRT1-knocked down HEK293T cells. (**M**) WB showed the levels of pT389-S6K, p-S6 and indicated proteins in NC or SIRT1 depleted HCT116 cells treated with indicated concentrations of BHB for 12 h. (**N**) Overexpressing SIRT1 in mouse colon organoids, and flow cytometry analysis of Ki-67^{+} frequency in mouse colon organoids treated with 1.0 mM BHB for 12 h (n = 3).

The statistical significance of the differences between groups was determined by (G to H, and N) two-way ANOVA or (K) one-way ANOVA (ns, not significant; *p < 0.05, **p < 0.01, ***p < 0.001). BHB denotes the D-isomer.

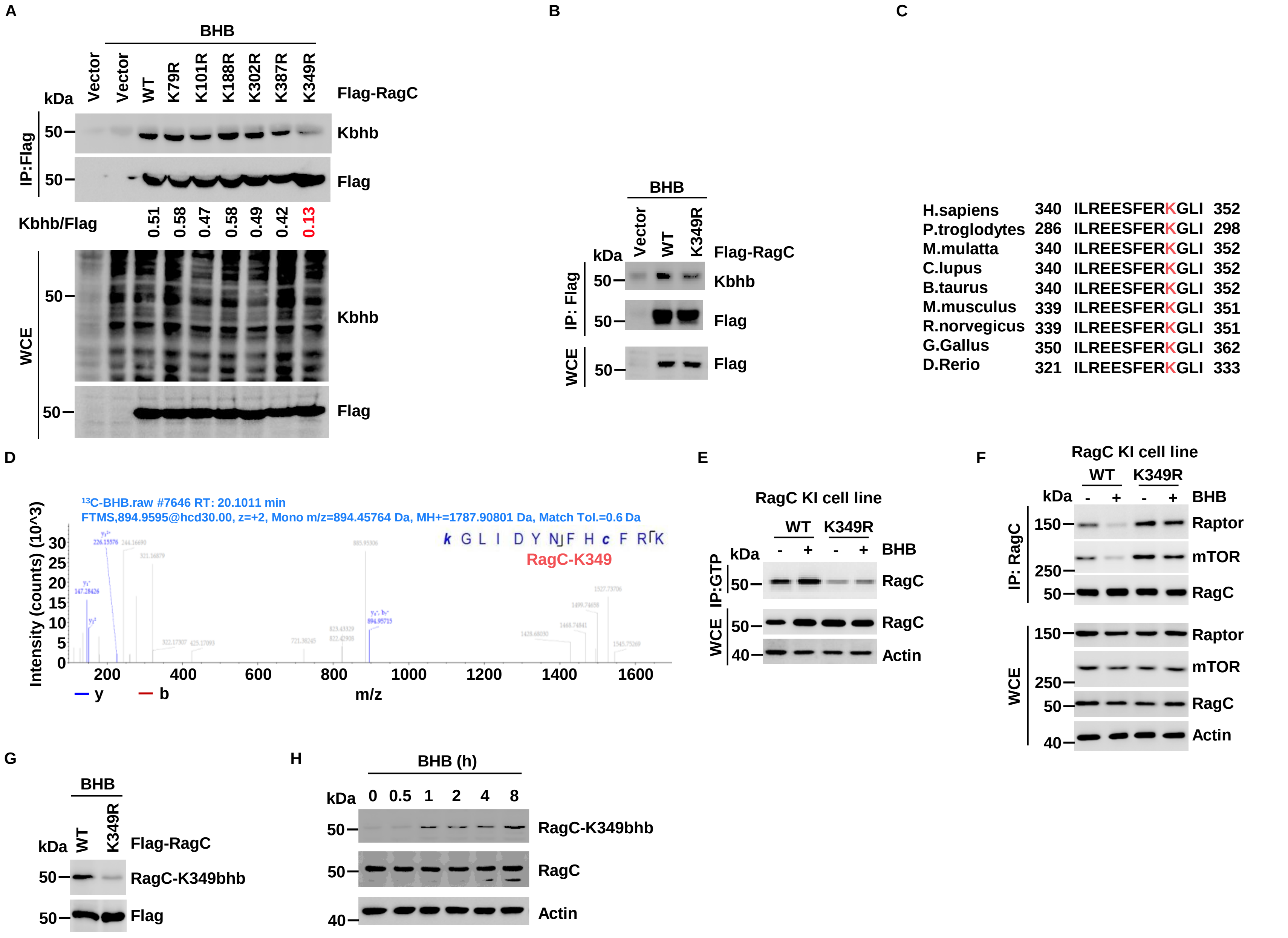

Figure S7. RagC is β-hydroxybutyrylated at K349.

(**A**) Measurement of Kbhb levels of RagC-WT, RagC-K79R, RagC-K101R, RagC-K188R, RagC-K302R, RagC-K387R and RagC-K349R after being treated with 1.0 mM BHB for 12 h. (**B**) Detecting RagC Kbhb in HEK293T cells transfected with RagC WT or K349R mutant plasmids and treated with 1.0 mM BHB for 12 h. (**C**) Sequence alignment of RagC in various species. red, Kbhb site. (**D**) MS/MS spectra from HPLC-MS/MS analysis of a β-hydroxybutyrylated peptide (the K349 site of human RagC) derived from HEK293T cells treated by 1.0 mM $^{13}C_4$ D-BHB. (**E**) Detecting the GTP-bound RagC using IP in RagC WT or K349R KI HCT116 cells treated with 1.0 mM BHB for 12 h. (**F**) IP showed interaction between RagC and Raptor/mTOR in RagC WT or K349R KI HCT116 cells treated with 1.0 mM BHB for 12 h. (**G**) Detecting RagC-K349bhb in whole lysates of HEK293T cells transfected with RagC WT or K349R mutant plasmids and treated with 1.0 mM BHB for 12 h. (**H**) Detecting RagC K349bhb in HCT116 cells treated with of 1.0 mM BHB for indicated time. BHB denotes the D-isomer.

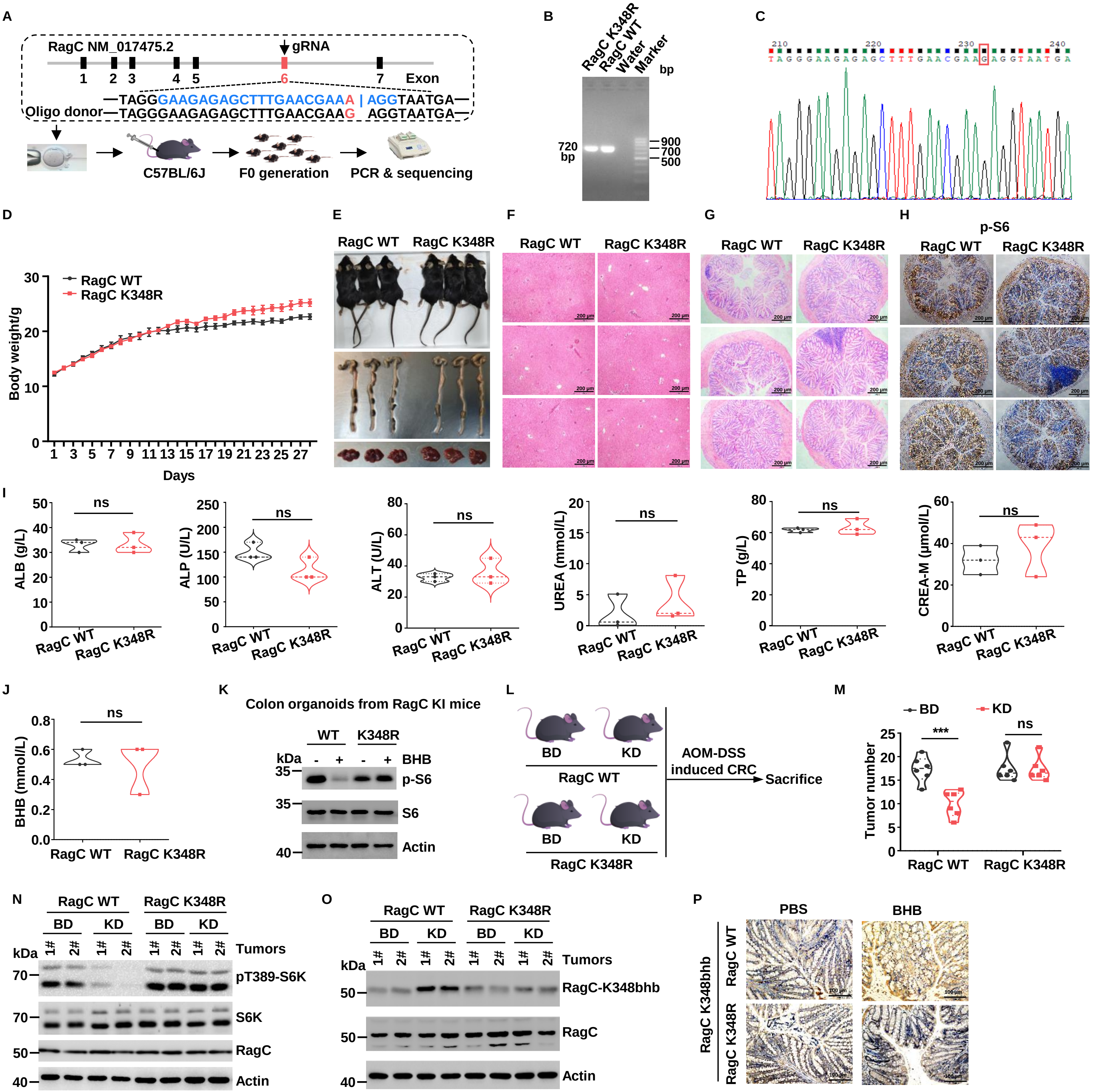

Figure S8. RagC-K349bhb inhibits colorectal tumors in mice.

(**A**) Methods and procedures for the construction of the RagC K348R KI mouse model. (**B** and **C**) Successful construction of a RagC K348R KI mouse model identified by PCR (B) and sequencing (C). (**D**) Growth rate of WT and RagC K348R KI mouse. (**E**) Appearance, liver, and colon morphology of WT and RagC K348R KI mouse. (**F**) Liver histological morphology of WT and RagC K348R KI mouse indicated by H&E staining, scale bar, 200 μm. (**G**) Colon histological morphology of WT and RagC K348R KI mouse indicated by H&E staining, scale bar, 200 μm. (**H**) The p-S6 levels of WT and RagC K348R KI mouse indicated by IHC, scale bar, 200 μm. (**I**) Serum ALB, ALP, ALT, UREA, TP and CREA concentration of WT and RagC K348R KI mouse (n = 3). (**J**) Serum BHB concentration of WT and RagC K348R KI mouse (n = 3). (**K**) Colon organoids derived from WT or KI mice were treated with 1.0 mM BHB for 12 h. WB analysis of the expression levels of p-S6, S6, and Actin across different experimental groups. (**L**) Experimental diagram for AOM/DSS-treated RagC WT or K348R KI mice fed KD or BD. (**M** to **O**) The tumors number (M), the level of pT389-S6K (N) and RagC-K348bhb (O) in tumor tissues of different groups was measured. (**P**) RagC WT or K348R KI mice subjected to AOM/DSS treatment received intraperitoneal injections of BHB, and the level of RagC-K348bhb in tumor tissues of different groups were measured.

The statistical significance of the differences between groups was determined by (I and J) unpaired two-tailed Student's t test or (M) two-way ANOVA (ns, not significant; ***p < 0.001). BHB denotes the D-isomer.
